# Jasmonate signalling pathway in strawberry: Genome-wide identification, molecular characterization and expression of *JAZ*s and *MYC*s during fruit development and ripening

**DOI:** 10.1371/journal.pone.0197118

**Published:** 2018-05-10

**Authors:** Adrián Garrido-Bigotes, Nicolás E. Figueroa, Pablo M. Figueroa, Carlos R. Figueroa

**Affiliations:** 1 Phytohormone Research Laboratory, Institute of Biological Sciences, Universidad de Talca, Talca, Chile; 2 Doctorate Program in Forest Sciences, Faculty of Forest Sciences, Universidad de Concepción, Concepción, Chile; Instituto de Biologia Molecular y Celular de Plantas, SPAIN

## Abstract

Jasmonates (JAs) are signalling molecules involved in stress responses, development and secondary metabolism biosynthesis, although their roles in fleshy-fruit development and ripening processes are not well known. In strawberry fruit, it has been proposed that JAs could regulate the early development through the activation of the JAs biosynthesis. Moreover, it has been reported that JA treatment increases anthocyanin content in strawberry fruit involving the bioactive jasmonate biosynthesis. Nevertheless, JA signalling pathway, of which main components are the COI1-JAZ co-receptor and the MYC transcription factors (TFs), has not been characterized in strawberry until now. Here we identified and characterized the woodland strawberry (*Fragaria vesca*) JAZ and MYC genes as well as studied their expression during development and ripening stages in commercial strawberry (*Fragaria* × *ananassa*) fruit. We described twelve putative JAZ proteins and two MYC TFs, which showed high conservation with respect to their orthologs in *Arabidopsis thaliana* and in other fleshy-fruit species such as *Malus* × *domestica*, *Vitis vinifera* and *Solanum lycopersicum* as revealed by gene synteny and phylogenetic analyses. Noteworthy, their expression levels exhibited a significant decrease from fruit development to ripening stages in *F*. × *ananassa*, along with others of the JA signalling-related genes such as *FaNINJA* and *FaJAM*s, encoding for negative regulators of JA responses. Moreover, we found that main JA signalling-related genes such as *FaMYC2*, and *FaJAZ1* are promptly induced by JA treatment at early times in *F*. × *ananassa* fruit. These results suggest the conservation of the canonical JA signalling pathway in strawberry and a possible role of this pathway in early strawberry fruit development, which also correlates negatively with the beginning of the ripening process.

## Introduction

Jasmonates (JAs) regulate development, metabolism and tolerance against biotic and abiotic stresses [1–3]. Their roles have not been extensively studied in fleshy fruits, although several reports have shown a role as stimulants of the phenylpropanoid pathway and ethylene biosynthesis [4]. Moreover, JAs and related oxylipins could play an early role during strawberry and grape fruit development since 12-oxo-phytodienoic acid (OPDA), methyl jasmonate (MeJA), jasmonic acid (JA), and the bioactive JA jasmonoyl-isoleucine (JA-Ile) accumulate at flowering and immature fruit stages, and then decrease as fruit ripens [5–7]. JAs are also involved in the anthocyanin accumulation in Arabidopsis seedlings [8] and exogenous MeJA application induces anthocyanin biosynthesis with the concomitant upregulation of JA biosynthesis-related genes in Chilean strawberry (*Fragaria chiloensis*) fruit [9]. In commercial strawberry (*Fragaria* × *ananassa*) fruit, after exogenous MeJA application, and coincident with the anthocyanin accumulation, JA-Ile levels increased along with anthocyanin accumulation, while the main ripening-associated hormone abscisic acid (ABA) decreased in developing treated fruit [7]. Recently, we characterized the dynamics of endogenous JAs during *F*. × *ananassa* fruit development and ripening [7]. A correlation between reduction of JA-Ile levels and downregulation of *FaJAR1*, the key gene encoding for the JA-Ile synthesis enzyme, and the JA turnover-related genes (i.e, *FaMJE* and *FaJIH1*) was reported [7]. Nevertheless, the molecular characterization of the JA signalling-related components has not been performed in strawberry until now.

The physiological effects mediated by JA-Ile require activation of the signalling pathway, which has been well characterized in Arabidopsis [2,10]. The F-box CORONATINE INSENSITIVE1 protein (COI1) is part of the Skp-Cullin-F-box-type E3 ubiquitin ligase complex (SCF^COI1^) and together with JASMONATE ZIM-DOMAIN (JAZ) form the JA-Ile receptor [11–13]. When JA-Ile levels are low, JAZ transcriptional repressors bind to MYC2 and additional transcription factors (TFs) repressing expression of early JA-responsive genes [2,10]. Moreover, Novel Interactor of JAZ (NINJA) adaptor allows the establishment of the co-repressor complex consisting of TOPLESS (TPL) [14] and histone deacetylases (HDAs) [15]. Once JA-Ile level rises, COI1 binds to JAZs that are degraded by the 26S proteasome after ubiquitination [2,10]. Then MYC2 and additional TFs induce the expression of early JA-responsive genes such as *JAZ*s, *MYC*s and JA biosynthetic ones [10,11,16]. In Arabidopsis, 13 JAZ proteins have been identified until now [10]. JAZ1-12 contain the conserved TIFY and Jas domains where JAZ13 is a non-TIFY JAZ protein [17]. The TIFY domain near N-terminal region contains the TIF[F/Y]XG motif [11,13,18], which mediates homo- and heteromeric interactions between TIFY proteins [19] and the interaction with NINJA [14]. The Jas domain is a conserved sequence at the C-terminal region of JAZ containing a conserved SLX_2_FX_2_KRX_2_RX_5_PY motif, mediating the hormone-dependent COI1-JAZ complex formation [12]. The Jas domain is also responsible for the interaction of JAZs with MYCs, suppressing JA responses [20]. On the other hand, PEAPOD (PPD) subfamily is part of the TIFY family proteins, which contain a highly conserved TIFY domain, a degenerated Jas domain and an N-terminal PPD domain. However, until now a PPD putative role in JA response has not been described [18,21,22]. MYC2, MYC3, MYC4 and MYC5 are bHLH-like TFs that contain a basic helix-loop-helix (bHLH) domain for binding to G-box-containing promoters [23–25] and the transcription is regulated by MED25 subunit [26]. MYC2, MYC3 and MYC4 interact with JAZs through the JAZ-interacting domain (JID) [27]. Other components of JA signalling pathway are JASMONATE-ASSOCIATED MYC2-like1/2/3 (JAM1, JAM2 and JAM3), which are antagonistic and negative regulators of MYC-like TFs and JA responses [28,29]. JAM1, JAM2 and JAM3 corresponding to bHLH003, bHLH013 and bHLH017 TFs, respectively [30]. Therefore, JAZs together with MYCs are the main regulators for JA-Ile responses [11] including upregulation of *JAZ* genes [11,24], which encoded JAZ proteins perform a negative feedback to turn off JA-Ile-mediated responses [11]. Recently, in a transcriptome analysis performed in *F*. × *ananassa* fruit, the repression of a *JAZ* from green to white and partially ripe stages was reported [31]. Nevertheless, a deep molecular characterization of the *JAZ* gene family and JA signalling components has not been reported in *Fragaria* species.

Thus, in this work we characterized the main JA signalling components in *Fragaria* species as JAZs and MYCs, using woodland strawberry (*Fragaria vesca*) to perform the genomic *in silico* studies and *F*. × *ananassa* for transcriptional analysis during fruit development and ripening and in response to JA treatment.

## Materials and methods

### Identification and characterization of JA signalling-related genes in *F*. *vesca*

Arabidopsis sequences of JA signalling-related proteins, including JAZs and MYCs, were used to search ortholog genes in *F*. *vesca* genome and transcriptome [32] by tblastn search tool (https://blast.ncbi.nlm.nih.gov/Blast.cgi) (S1 Table). Sequences with higher coverage and identity, and with lower e-value were selected for further bioinformatics analysis (S1 Table). *TIFY5b-like* sequence (GenBank accession number XM_011469361.1) was obtained directly from *F*. *vesca* transcriptome because it showed lower coverages, identities and higher e-values comparing with Arabidopsis JAZ proteins.

### Mapping, duplication and synteny analysis of *JAZ* and *MYC* genes in *F*. *vesca* and *Arabidopsis thaliana*

Physical chromosomal locations of *F*. *vesca* (Fv) and *Arabidopsis thaliana* (At) *JAZ*s and *MYC*s were obtained from Arabidopsis (TAIR10, https://www.arabidopsis.org/) and Genome databases (https://www.ncbi.nlm.nih.gov/genome/). Gene synteny analysis were performed between the *F*. *vesca*, *Malus* × *domestica* (Md), *Vitis vinifera* (Vv), *Solanum lycopersicum* (Sl), *Oryza sativa* (Os) and *A*. *thaliana* genomes (S2 Table) using information available in the Plant Genome Duplication Database (PGDD) [33].

### Gene structure, protein sequence and phylogenetic analyses

Exon-intron organizations of *JAZ* and *MYC* genes were determined by Gene Display Server 2.0 [34] using the information available at Genome database (https://www.ncbi.nlm.nih.gov/genome). *F*. *vesca* and Arabidopsis JAZ and MYC TFs sequences were analyzed for structural and functional domains. *F*. *vesca* JAZs and MYCs encoding sequences were obtained from *F*. *vesca* transcriptome (https://www.ncbi.nlm.nih.gov/refseq) (S1 Table). TIFY and Jas domains were identified through multiple sequence alignment by Clustal Omega [35] and visualized by Jalview software [36]. Logo sequences of JAZ and MYCs domains were obtained using Weblogo 3 [37]. Unrooted phylogenetic trees were built using full-length amino acidic sequences by the neighbor-joining (NJ) method and a bootstrap of 1,000 replicates. Phylogenetic trees were visualized by Evolview tool [38]. We renamed the annotated *F*. *vesca* TIFY and MYC sequences according to their homology degree; length, domain location and clustering in phylogenetic tree with the corresponding Arabidopsis sequences.

### Plant material and JA treatment

Strawberry (*F*. × *ananassa* cv. Aromas) flowers and fruit were collected at different developmental stages from plants grown in a commercial field at Angol, Araucanía Region, Chile (latitude 37°45’18” S; longitude 72°36’49” W) during three different dates in the 2014 growing season. The owner of the land gave permission to conduct the study on this site. The picked flowers and fruit were transported to the laboratory under refrigerated conditions and classified in six developmental stages corresponding to 0 (flowering, F), 10 (small green, SG), 17 (large green, LG), 20 (white, W), 21 (turning, T), 23 (50% red receptacle, 50%R) and 25 (100% red receptacle, 100%R) days after anthesis (DAA) as previously reported [7].

On the other hand, we performed an experiment to verify JA treatment effects on *MYC2* and *JAZ*s gene expression during an *in vitro* fruit ripening system. Peduncles of three fruit at white stage (W) were trimmed to a uniform length of 50 mm and immersed in sterile tubes (50 ml) with autoclaved distilled water containing 88 mM sucrose and 1 mM hydroxyquinoline hemisulfate (HQS) plus 100 μM MeJA according to previously reported [7,9]. The fruit in solution were incubated in a growth chamber under standard fluorescent lights (16 h photoperiod and 40 μmol/m^2^.s^1^ light intensity) at 24°C. Fruit sampling was performed at 15 min, 30 min, 1 h and 6 h of MeJA incubation. At each treatment and time, three biological replicates were used for gene expression analysis.

### Gene expression analysis

Total RNA was isolated using the CTAB method [39] and mini-columns for RNA purification (RNeasy Plus Mini Kit, Qiagen, Germany). The cDNA synthesis was performed using the RevertAid H Minus First Strand cDNA Synthesis Kit (ThermoScientific, Finland) according to the manufacturer's instructions. Expression analysis of JA signalling-related genes was performed using reverse transcription-qPCR (RT-qPCR). Primer3 and Primer-BLAST tools using full-length CDS sequences of *F*. *vesca* as templates were utilized for primer design for each gene. Primers used for RT-qPCR are described in S3 Table and generated single products. *FvJAZ4-1*, *FvJAZ4-2* and *FvJAZ4-3* primers were designed in a shared sequence region by the three CDS. RT-qPCR was performed following the instructions of KAPA SYBR FAST qPCR kit (KAPA Biosystems, USA) according to the manufacturer's instructions in a PikoReal Real-Time PCR System (Thermo Scientific, Finland). The expression levels were calculated according to the 2^-ΔΔCT^ method [40] and expressed in relative arbitrary units, normalized according to housekeeping gene glyceraldehyde 3-phosphate dehydrogenase (*GAPDH*). The PCR conditions were as follows: 95°C for 10 min; 40 cycles of 95°C for 15 s, 60°C for 15 s and 72°C for 15 s; and a melting curve of 60°C for 30 s, 95°C for 15 s and 20°C for 10 min. All RT-qPCR reactions were performed with three biological and three technical replicates. The Heatmapper software [41] was used to estimate the fold-change of gene expression.

### Determination of anthocyanins and proanthocyanidins (PAs) contents

Five grams of frozen fruit skin tissue without achenes were grounded with liquid nitrogen, homogenized with 15 ml of acetone/water (80/20 v/v) and stored at -20°C until use. Anthocyanins quantification was performed according to previously reported [42]. Briefly, 50 μl of fruit extract and 150 μl of corresponding buffer were dispensed into a 96-well plate. The absorbance was measured at 509 nm and 700 nm, considering ε = 17,330 L/cm^1^.mol^1^. The results were expressed as mg of pelargonidin-3-glucoside equivalents per 100 g of fresh weight (FW). PAs content was measured according to previously reported [43]. Briefly, 70 μl of fruit extract diluted (1/10, v/v) and 210 μl of dimethylaminocinnamaldehyde (DMAC) reagent were dispensed into wells of a 96-well plate. The microplate was read for 20 min at 640 nm. The concentration was calculated from a calibration curve, using catechin as standard. The results were expressed as mg of catechin equivalents per 100 g of FW.

## Results

### Identification, chromosomal location and synteny of *JAZ* and *MYC* genes in *F*. *vesca*

In order to identify ortholog *JAZ* and *MYC* genes in *F*. *vesca*, a tblastn search was performed using Arabidopsis JAZs and MYCs protein sequences as queries (S1 Table). In *F*. *vesca*, we identified and named 12 non-redundant members (FvJAZ1, FvJAZ4-1, FvJAZ4-2, FvJAZ4-3, FvJAZ5, FvJAZ7, FvJAZ8.1, FvJAZ8.2, FvJAZ9, FvJAZ10, FvJAZ11 and FvJAZ12), which are annotated as TIFY proteins in *F*. *vesca* database (Table 1, S1 and S4 Tables). Due to the similar number of JAZs in *F*. *vesca* and Arabidopsis, we decided to name the *F*. *vesca* JAZ according to their well-studied Arabidopsis ortholog genes. Moreover, we identified two MYC transcription factors, FvMYC2 and FvMYC2-like, which are annotated as MYC2 and MYC2-like in *F*. *vesca* databases (Table 1, S1 and S5 Tables). However, Arabidopsis MYC3, MYC4 and MYC5 orthologs were not identified in *F*. *vesca* databases.

**Table 1:** Genomic data of *JAZ* and *MYC* gene family in woodland strawberry (*Fragaria vesca*) and Arabidopsis, and their corresponding CDS and protein lengths.

Gene name ^a^	Gene ID	Chromosome	Start	End	Gene (bp)	CDS (bp)	ORF (aa)
*FvJAZ1 / FvTIFY10A*	101302102	1	6964291	6965689	1349	909	302
*FvJAZ4-1 / FvTIFY6B*.*1*	101298700	4	19330995	19333777	1934	1158	385
*FvJAZ4-2 / FvTIFY6B*.*2*	101298700	4	19330995	19333777	1960	1155	384
*FvJAZ4-3 / FvTIFY6B*.*3*	101298700	4	19330995	19333777	1701	1083	360
*FvJAZ5 / FvTIFY11A-like*	101305492	6	24774113	24774773	1016	558	185
*FvJAZ7 / FvTIFY5B*	105352369	6	27798266	27799489	814	372	123
*FvJAZ8*.*1 / FvTIFY5A*	101295112	3	2612521	2613468	862	393	130
*FvJAZ8*.*2 / FvTIFY5B-like*	105350256	3	2616234	2616765	794	420	139
*FvJAZ9 / FvTIFY6B*	101303423	5	9961254	9964696	1678	1116	371
*FvJAZ10 / FvTIFY9*	101299545	1	1660803	1661807	1489	576	191
*FvJAZ11 / FvTIFY3A-like*	105349490	Unknown	159768	161085	561	561	186
*FvJAZ12 / FvTIFY3B*	101312185	1	7358182	7359713	1172	609	202
*FvMYC2*	101299702	7	2955350	2956825	1955	1476	491
*FvMYC2-like*	101308180	5	21462454	21464502	2690	2049	682
*AtJAZ1 / AtTIFY10A*	AT1G19180	1	6622312	6623271	1892	762	253
*AtJAZ2 / AtTIFY10B*	AT1G74950	1	28148919	28150258	1873	750	249
*AtJAZ3 / AtTIFY6B*	AT3G17860	3	6119968	6122691	3338	1059	352
*AtJAZ4 / AtTIFY6A*	AT1G48500	1	17931658	17934255	3273	834	310
*AtJAZ5 / AtTIFY11A*	AT1G17380	1	5955654	5957070	2357	825	274
*AtJAZ6 / AtTIFY11B*	AT1G72450	1	27274336	27276136	2595	810	269
*AtJAZ7 / AtTIFY5B*	AT2G34600	2	14573172	14573718	923	447	148
*AtJAZ8 / AtTIFY5A*	AT1G30135	1	10596516	10597095	990	396	131
*AtJAZ9 / AtTIFY7*	AT1G70700	1	26654951	26656804	2822	804	243
*AtJAZ10 / AtTIFY9*	AT5G13220	5	4219001	4220502	2292	504	197
*AtJAZ11 / AtTIFY3A*	AT3G43440	3	15367670	15369774	2612	717	238
*AtJAZ12 / AtTIFY3B*	AT5G20900	5	7090883	7092201	1836	564	187
*AtJAZ13*	AT3G22275	3	7878807	7879810	827	378	125
*AtMYC2*	AT1G32640	1	11799042	11800913	3289	1872	623
*AtMYC3*	AT5G46760	5	18974231	18976009	2569	1779	592
*AtMYC4*	AT4G17880	4	9933702	9935471	2360	1770	589
*AtMYC5*	AT5G46830	5	19002719	19004254	1821	1536	511

^a^ *F*. *vesca* (*Fv*) and Arabidopsis (*At*) gene sequences were obtained from the National Center for Biotechnology Information NCBI, https://www.ncbi.nlm.nih.gov/genome) and Arabidopsis database (TAIR10, http://www.arabidopsis.org), respectively.

**Fig 1:**
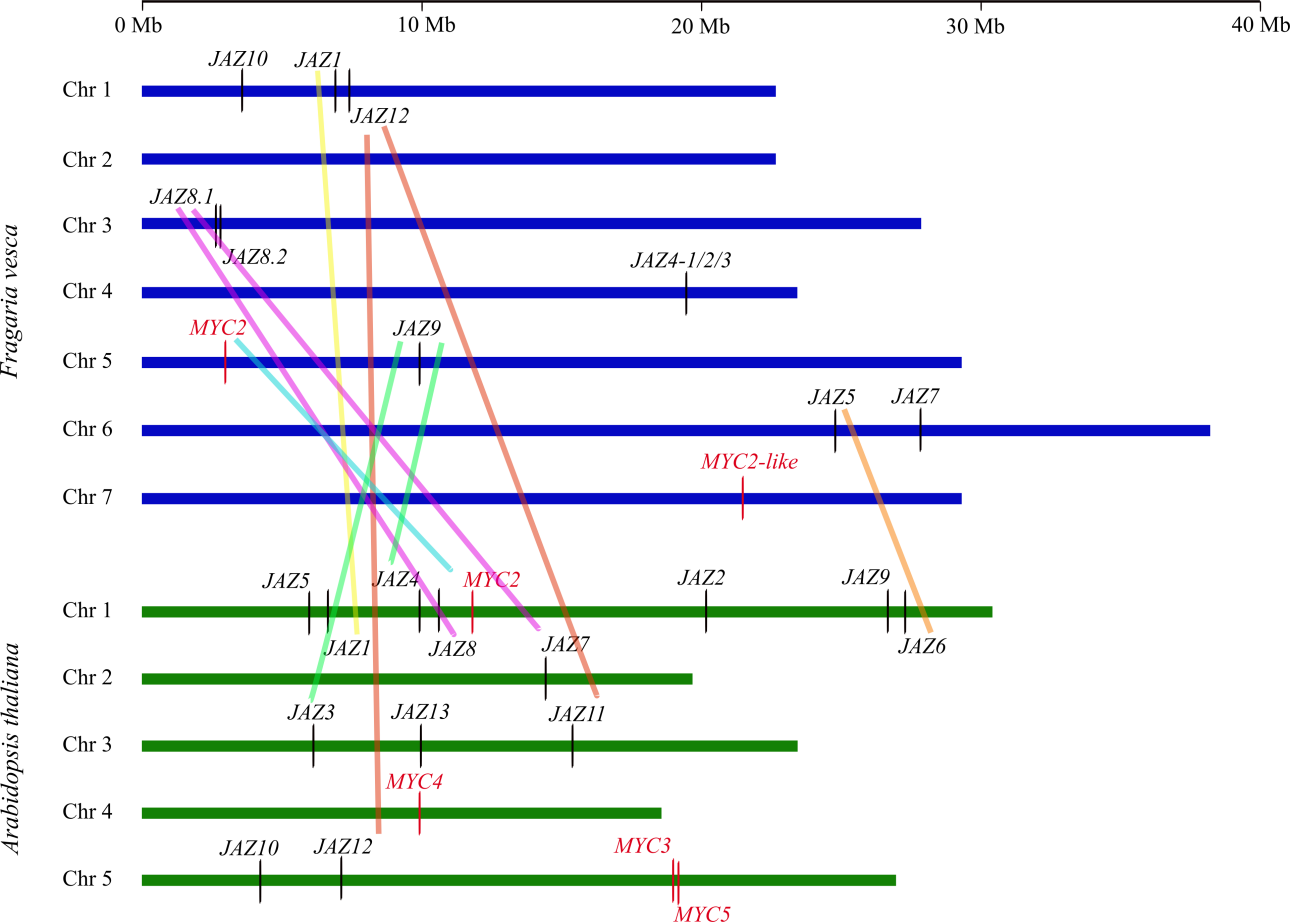
Genome distribution and synteny of *JAZ* and *MYC* genes in *Fragaria vesca* and Arabidopsis chromosomes. Chromosomes are indicated as horizontal blue and green bars. *JAZ*s are indicated by black letters and vertical lines and *MYC*s*-like* are indicated by red letters and vertical lines. Thick colored lines denote syntenic regions. *FvJAZ11* has unknown location. JAZ, jasmonate ZIM-domain.

Next, we compared chromosomal locations of *JAZ* and *MYC* genes and constructed a schematic representation of their distribution (Fig 1) based on information available from *F*. *vesca* and Arabidopsis genome databases (Table 1 and S2 Table). The *FvJAZ* genes were located on chromosomes 1, 3, 4, 5 and 6, while *FvMYC2* and *FvMYC2-like* did on chromosomes 5 and 7, respectively (Fig 1, Table 1 and S2 Table). However, *FvJAZ11* could not be located in *F*. *vesca* genome (Fig 1, Table 1 and S2 Table), likely because of *F*. *vesca* genome is not completely assembled (https://www.ncbi.nlm.nih.gov/genome/3314). Additionally, we searched and detected conserved syntenic regions corresponding to segmental duplications between *JAZ* and *MYC* genes of *F*. *vesca* and Arabidopsis using the Plant Genome Duplication Database (PGDD). *FvJAZ1*, *FvJAZ8*.*1* and *FvJAZ12* exhibited synteny with their Arabidopsis ortholog genes and *FvJAZ8*.*1* and *FvJAZ12* showed an additional synteny with near ortholog genes *AtJAZ7* and *AtJAZ11*, respectively (Fig 1 and S1 Table). We found a tandemly duplication in *F*. *vesca* genome corresponding to *FvJAZ8*.*1* and *FvJAZ8*.*2* genes (Fig 1 and S1 Table). *FvJAZ5* and *FvJAZ9* showed synteny with *AtJAZ6* and *AtJAZ3* and *AtJAZ4*, respectively (Fig 1). Other genes such as *FvJAZ4-1*, *FvJAZ4-2*, *FvJAZ4-3*, *FvJAZ7*, *FvJAZ8*.*2*, *FvJAZ10* and *FvMYC2-like* could not be mapped to syntenic regions into Arabidopsis genome (Fig 1). *FvMYC2* showed synteny with its *AtMYC2* ortholog gene (Fig 1 and S1 Table). Moreover, chromosome location and synteny analysis of *M*. × *domestica*, *S*. *lycopersicum*, *V*. *vinifera* and *O*. *sativa JAZ* and *MYC*-*like* genes was performed respect *A*. *thaliana* orthologs, using information available in PGDD and species-genomic databases (S1 Fig and S2 Table). *V*. *vinifera* displayed higher number of *JAZ* and *MYC-like* syntenic genes respect to *A*. *thaliana* genes (S1 Fig). An additional non-annotated *VvMYC2-like* gene showed synteny with *AtMYC2* (S1 Fig). In the case of *M*. × *domestica*, *S*. *lycopersicum* and *O*. *sativa*, only four syntenic regions were detected for each one (S1 Fig). In summary, *FvJAZ* and *FvMYC* are orthologs and syntenic genes respect to *A*. *thaliana* ones, similarly to *M*. × *domestica*, *V*. *vinifera*, *S*. *lycopersicum* and *O*. *sativa*.

### Exon-intron structure analysis for *JAZ* and *MYC* genes in *F*. *vesca*

**Fig 2:**
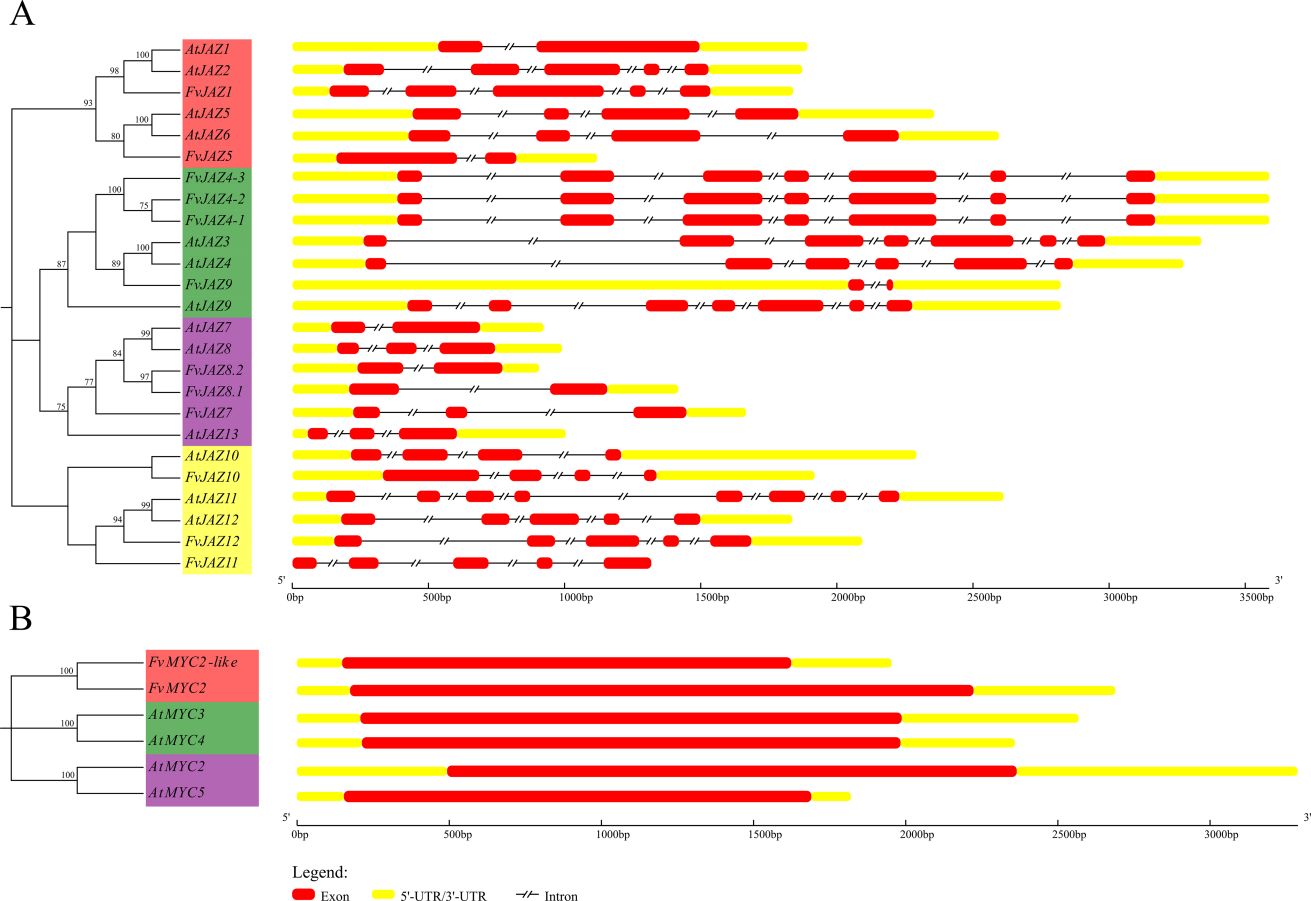
Exon-intron structures of the *Fragaria vesca* and Arabidopsis *JAZ* and *MYC* genes. Exon-intron organization of *JAZ* genes (A) and *MYC* genes (B) in *F*. *vesca* (Fv) and *Arabidopsis thaliana* (At) grouped according their gene orthology. Yellow and red bars indicate untranslated (UTR) regions and exons, respectively. Black interrupted lines indicate introns. JAZ, jasmonate ZIM-domain.

In order to gain insights into the diversification of the *JAZ*s and *MYC*s, we compared exon-intron organization among all genes of *F*. *vesca* and Arabidopsis (Fig 2). We observed that *FvJAZs* and *AtJAZ*s contain a variable intron numbers (Fig 2A); in contrast, all *FvMYC* and *AtMYC* genes lack introns (Fig 2B). We detected variations in length and number of introns in *FvJAZ* genes. *FvJAZ10* and *FvJAZ12* maintained the number of introns, although with length variations, with respect to Arabidopsis orthologs (Fig 2A). Other *FvJAZ* genes such as *FvJAZ4*s, *FvJAZ5*, *FvJAZ7*, *FvJAZ8*.*1*, *FvJAZ8*.*2* and *FvJAZ9* showed variable numbers of introns from one to six (Fig 2A). On the other hand, *FvJAZ1* gained three introns and *FvJAZ4*s and *FvJAZ7* genes gained one intron each one; while *FvJAZ5*, *FvJAZ8*.*1*, *FvJAZ9* and *FvJAZ11* lost two, one, five and three introns, respectively, regarding their Arabidopsis ortholog genes (Fig 2A). Moreover, *FvMYC2* and *FvMYC2-like* lack introns (Fig 2B) as well as *A*. *thaliana* orthologs. Overall, some *FvJAZ* genes display a variable exon-intron organization, while *FvMYC2* and *FvMYC2*-like genes keep the lack of introns.

### Conserved domains of JAZ and MYC proteins in *F*. *vesca*

**Fig 3:**
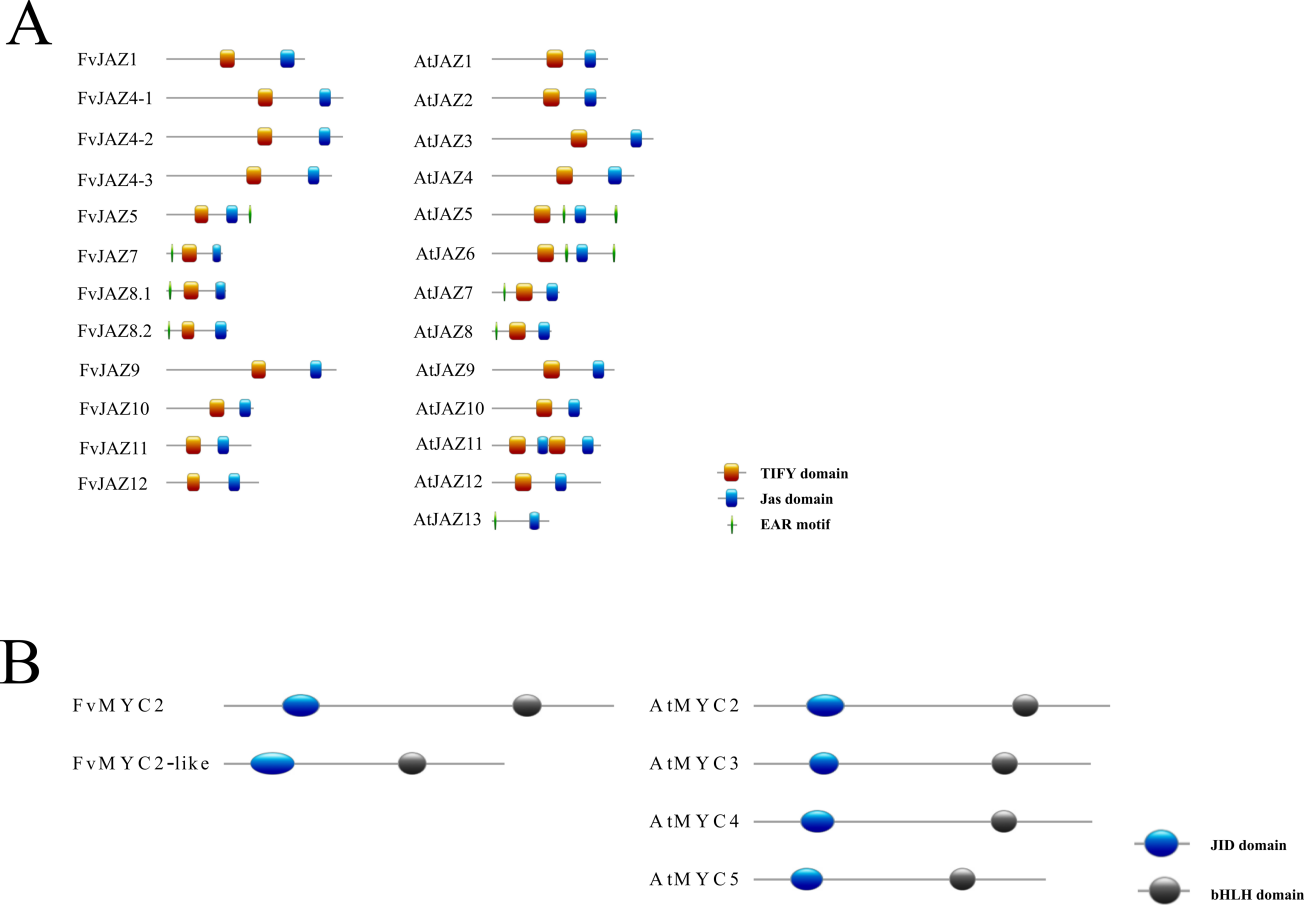
Distribution of JAZ and MYC protein domains and motifs in *Fragaria vesca* and Arabidopsis. Comparative distribution of TIFY, Jas and EAR domains and motifs in JAZ proteins (A) and comparative distributions of JID and bHLH domains in MYC-like proteins (B) of *Fragaria vesca* (Fv) and *Arabidopsis thaliana* (At). The relative position of each domain within each protein are displayed in colors. bHLH, basic helix-loop-helix; EAR, ethylene-responsive element binding factor-associated amphiphilic repression; JAZ, jasmonate ZIM-domain; JID, JAZ-interacting domain.

**Fig 4:**
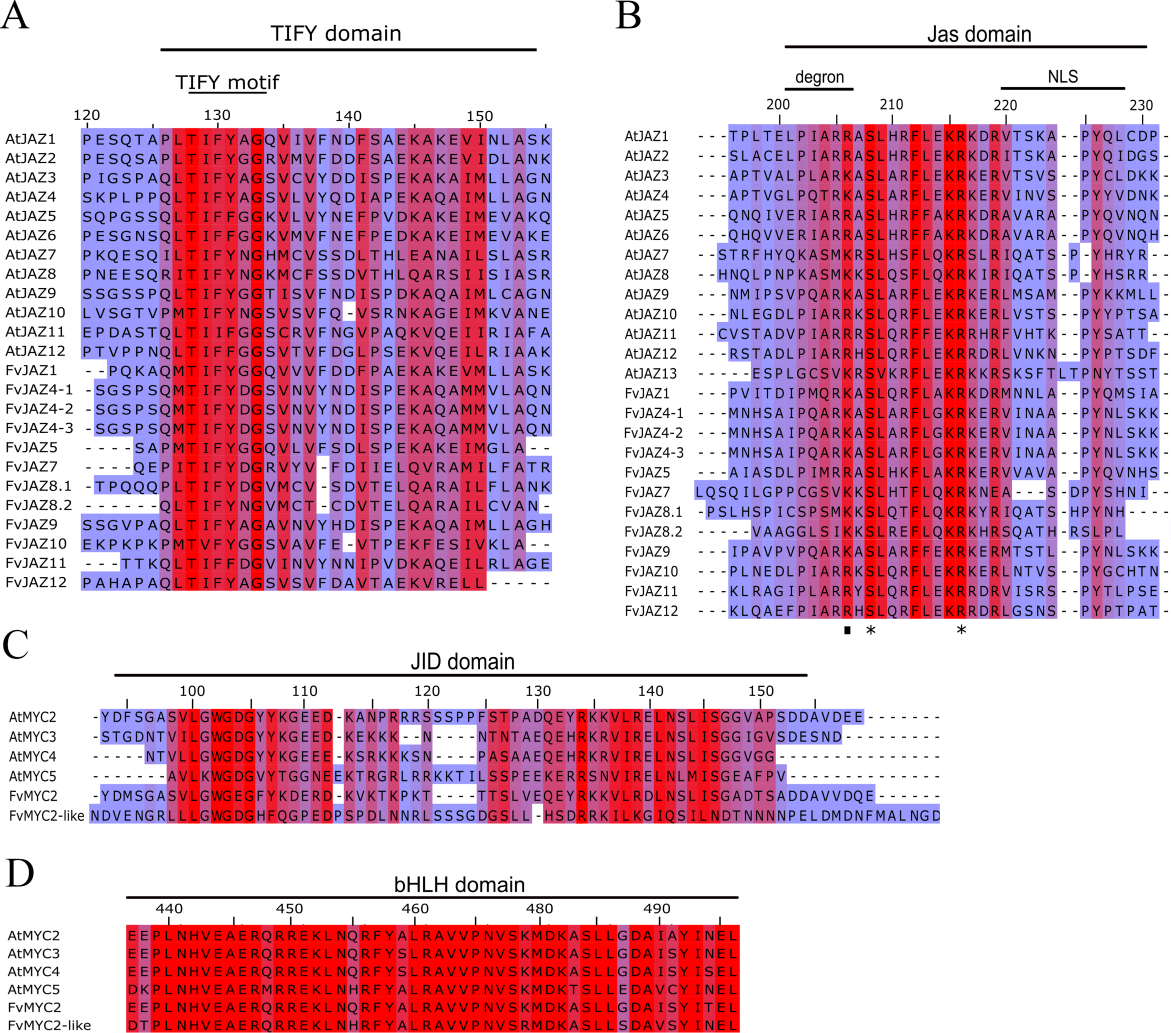
Multiple alignment of JAZ and MYC protein domains in *Fragaria vesca* and Arabidopsis. Multiple sequence alignment of TIFY (A), Jas (B), JID (C) and bHLH (D) domains of Arabidopsis and putative *F*. *vesca* JAZ and MYC sequences. Red and blue colors indicate higher and lower amino acidic residues conservation, respectively. Dot and asterisks indicate conserved residues involved in interaction with COI1 and MYCs, respectively. bHLH, basic helix-loop-helix; JAZ, jasmonate ZIM-domain; JID, JAZ-interacting domain; NLS, nuclear localization signal.

**Fig 5:**
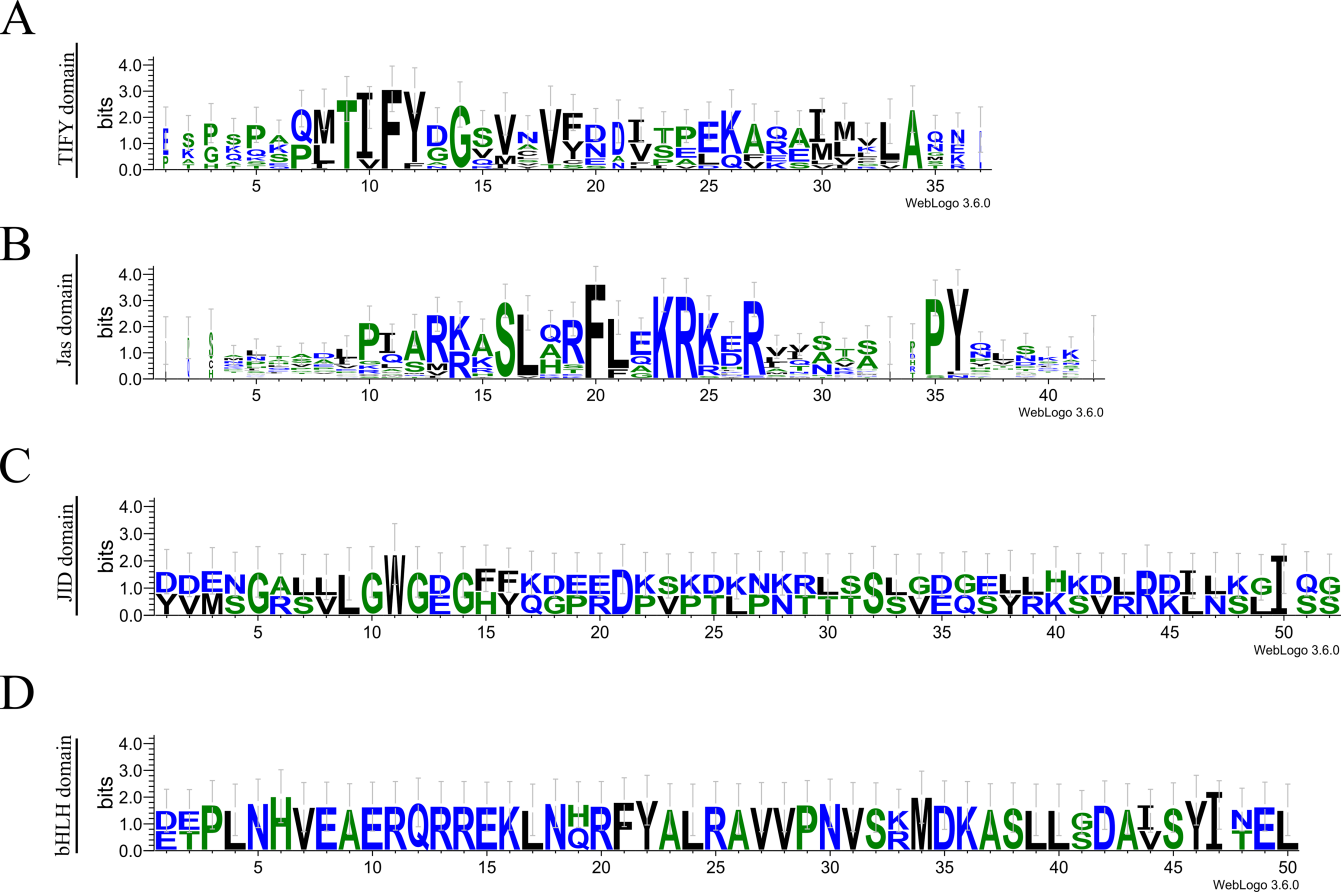
Logo sequences for FvJAZ and FvMYC proteins. Logo sequences for TIFY (A), and Jas (B) domains of FvJAZ proteins, and JID (C) and bHLH (D) domains of FvMYC proteins. bHLH, basic helix-loop-helix; JID, JAZ-interacting domain; JAZ, jasmonate ZIM-domain.

To confirm evolutionary relationships and gain further insights in the primary protein structure between JAZ proteins, the domain and motifs positions were evaluated (Figs 3 and 4). FvJAZ proteins showed a length range from 123 to 385 amino acid residues according to that observed in Arabidopsis (Table 1 and S6 Table). The distribution of domains is similar between protein sequences of *F*. *vesca* and their orthologs in Arabidopsis (Fig 3A). Analysis of the deduced amino acid sequence of *F*. *vesca* JAZ showed the conservation of the TIFY/ZIM and Jas domains (Fig 4A and 4B). In *F*. *vesca*, the TIFY domain displayed the highly conserved TIFY[F/Y]XG motif (Fig 4A), although with TVFYXG and TIFFXG variants present in FvJAZ10 and FvJAZ11, respectively (Fig 4A). Jas domain displayed conserved sequence SLX2FLXKR[K/R]X[R/E] like consensus sequence in *F*. *vesca* (Fig 4B). Although some proteins like FvJAZ7, FvJAZ8.1 and FvJAZ8.2 showed a variant Jas sequence (Fig 4B and S3 Fig). FvJAZ10 exhibited the canonical degron LPIARK whereas other *F*. *vesca* JAZ proteins exhibited variations in degron amino acid residues such as IPMQRK in FvJAZ1, IPQARK in FvJAZ4s, LPIMRR in FvJAZ5, VPQARK in FvJAZ9, IPLARR in FvJAZ11 and FPIARR in FvJAZ12 (Fig 4B). FvJAZ11 did not show the duplicated TIFY and Jas domains that are present in AtJAZ11 (Fig 3A). On the other hand, C-terminal X5PYX2 region, which may act as Nuclear Localization Signal (NLS) exhibited conservation among JAZ proteins of *F*. *vesca* (Fig 4B). In this regard, FvJAZ5 showed an EAR-motif at the C-terminus, while FvJAZ7, FvJAZ8.1 and FvJAZ8.2 displayed this motif at the N-terminus (Fig 3A and S2 Fig). In the case of FvJAZ5, we noticed that it lacks the DLNEPT motif or similar sequence (Panel A in S2 Fig). TIFY and Jas domain logo sequences showed a highly residue conservation in FvJAZ proteins (Fig 5A and 5B). On the other hand, domain structure and sequences analysis of FvMYC TFs were evaluated and compared with their Arabidopsis orthologs (Figs 3B, 4C and 4D). FvMYC2 and FvMYC2-like showed two conserved domains corresponding to JID and bHLH and displayed conserved position and different domain lengths (Figs 3B, 4C and 4D). Deduced consensus sequences of FvMYC2 and FvMYC2-like JID domain showed conservation between some amino acidic residues (Fig 4C), while bHLH domain displayed highly similarity between MYC-like proteins (Fig 4D). FvMYC2-like contain amino acidic residues more similar to AtMYC5 (Fig 4D). The logo sequences of JID and bHLH domains indicated high conservation of residues in FvMYC proteins (Fig 5C and 5D). Globally, FvJAZs and FvMYCs contain domains highly conserved, with similar domain locations and protein lengths.

### Phylogenetic analysis of *F*. *vesca* JAZ and MYC2-like proteins

**Fig 6:**
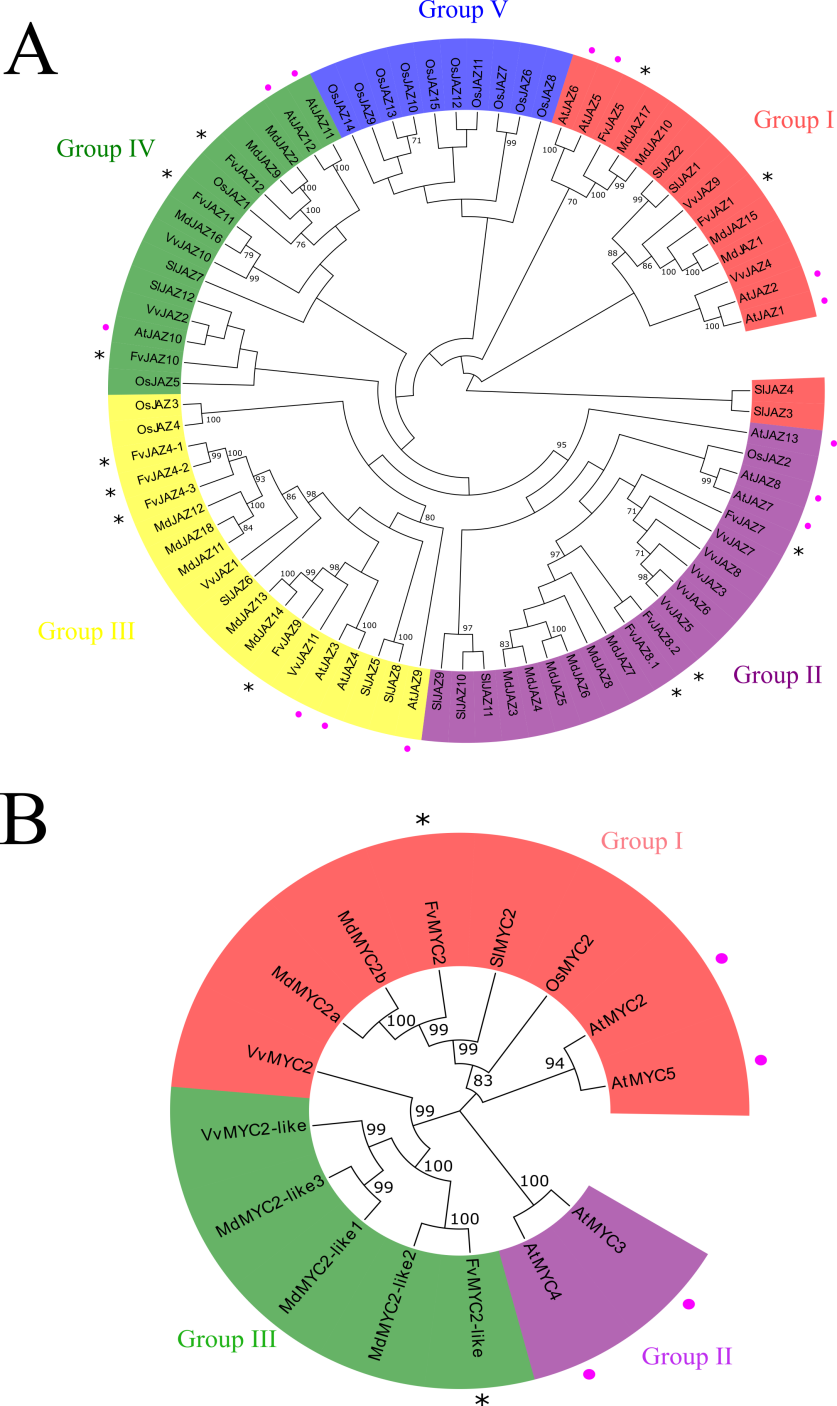
Phylogenetic analysis of *Fragaria vesca* JAZ and MYC proteins. Phylogenetic tree of JAZ (A) and MYC-like proteins sequences (B) from *Fragaria vesca* (Fv), *Arabidopsis thaliana* (At), *Malus* × *domestica* (Md), *Vitis vinifera* (Vv), *Solanum lycopersicum* (Sl) and *Oryza sativa* (Os). The phylogenetic analysis was performed using full-length JAZ and MYC protein sequences. FvJAZ and FvMYC2-like, and AtJAZ and AtMYC-like proteins are indicated by asterisk and pink dots, respectively. Nodes with bootstrap values > 70% are labelled and bootstrap values ≥ 95% show highlight bootstrap. JAZ, jasmonate ZIM-domain.

Unrooted phylogenetic trees were generated by neighbor-joining (NJ) algorithm by bootstrap of 1,000 replicates, showing evolutionary relationships between the JAZ and MYC2-like proteins of the dicot plants such as *A*. *thaliana*, *F*. *vesca*, *M*. × *domestica*, *V*. *vinifera*, *S*. *lycopersicum* and the monocot *O*. *sativa*. JAZ proteins were clustered in five groups (Fig 6). Regarding to the JAZ protein subfamilies, FvJAZ1 and FvJAZ5 were clustered together with AtJAZ1, AtJAZ2, AtJAZ5 and AtJAZ6 proteins into group I together some *M*. × *domestica*, *V*. *vinifera* and *S*. *lycopersicum* ortholog proteins (Fig 6A) and showed 40.1–43.9% identity with the *A*. *thaliana* orthologs AtJAZ1 and AtJAZ5, respectively (S4 Table). Otherwise, in group II, FvJAZ7, FvJAZ8.2 and FvJAZ8.1 clustered together (Fig 6A) and showed between 36.8–47% identity with their corresponding *A*. *thaliana* orthologs (S4 Table), and clustered in the same group of the non-TIFY protein AtJAZ13 and *M*. × *domestica*, *V*. *vinifera*, *S*. *lycopersicum* JAZs and OsJAZ2 proteins (Fig 6A). On the other hand, FvJAZ4s and FvJAZ9 were grouped into group III together with AtJAZ3, AtJAZ4 and AtJAZ9 including JAZ proteins from other species (Fig 6A). Finally, *A*. *thaliana* and *F*. *vesca* JAZ10, JAZ11 and JAZ12 proteins were clustered in the group IV (Fig 6A). Nevertheless, FvJAZ10 and FvJAZ11 showed higher similarity with AtJAZ4 on the sequence identity matrix (S4 Table), which was not reproduced in the phylogenetic tree position (Fig 6A). Additionally, some *O*. *sativa* JAZ proteins were clustered together in group V displaying divergence with dicots-associated JAZs (Fig 6A). Bootstrap values showed (> 70%) indicated high reliability in most clusters (Fig 6A), and values ≥ 95% represented groupings with the higher confidence.

To analyze the evolutionary relationships between MYC-like TFs of *F*. *vesca*, *A*. *thaliana*, *M*. × *domestica*, *V*. *vinifera*, *S*. *lycopersicum* and *O*. *sativa*, an unrooted phylogenetic tree was performed (Fig 6B). FvMYC2 showed the highest identity (58.5%) with AtMYC2 (S5 Table) and it was clustered in the group I close to AtMYC2 in the phylogenetic tree (Fig 6B). Other MYC2 TFs corresponding to *M*. × *domestica*, *S*. *lycopersicum* and *O*. *sativa* were also clustered in this group (Fig 6B). On the other hand, FvMYC2-like was grouped into group II along with VvMYC2-like TFs (Fig 6B). AtMYC3 and AtMYC4 were clustered together in a group III (Fig 6B). Phylogenetic tree nodes showed bootstrap values ≥ 95%, except for OsMYC2, exhibiting high confidence levels (Fig 6B). In summary, FvJAZ, FvMYC2 and FvMYC2-like proteins share high similarity and show closer evolutionary relationships within dicots (*F*. *vesca*, *A*. *thaliana*, *M*. × *domestica*, *S*. *lycopersicum* and *V*. *vinifera*).

### Expression of *FaJAZ* and *FaMYC* genes during development and ripening of *F*. × *ananassa* fruit

**Fig 7:**
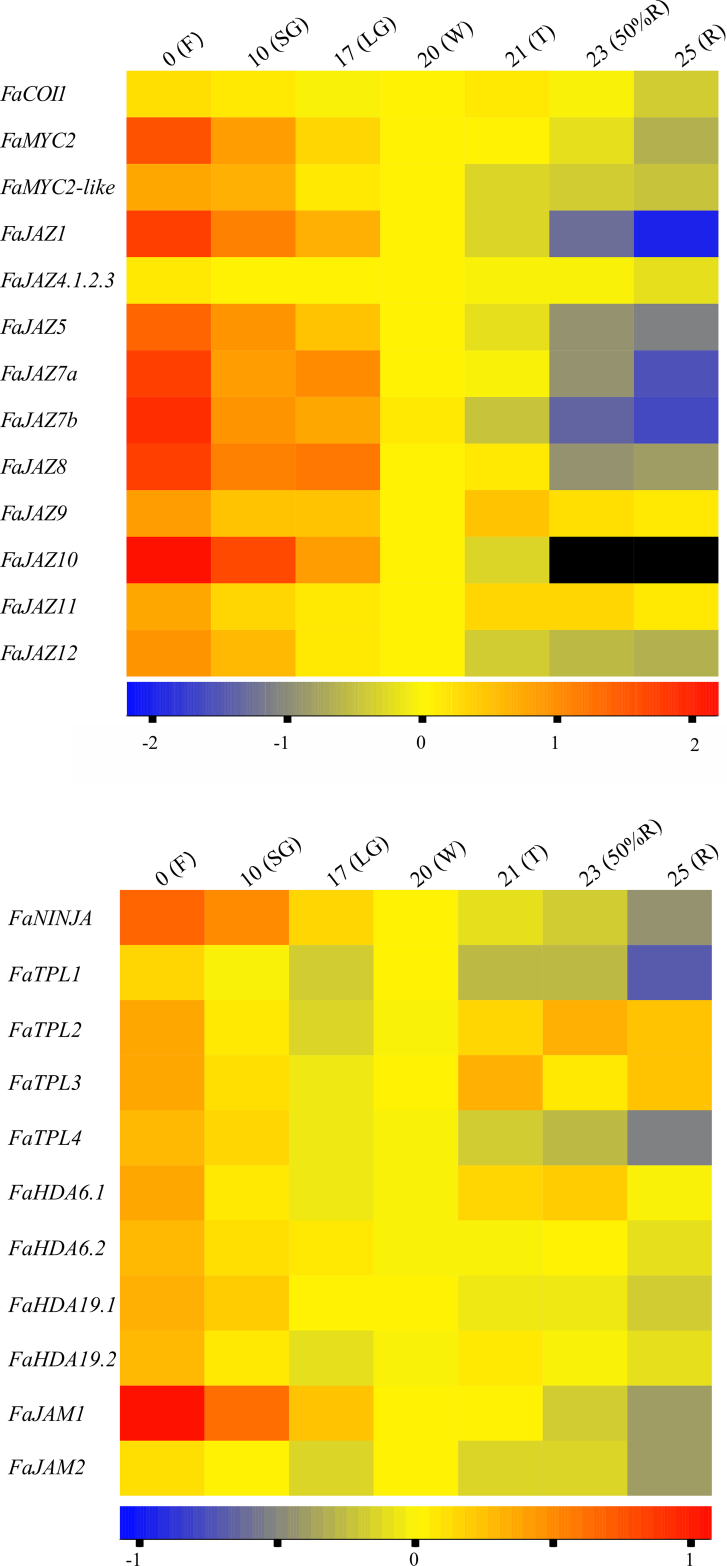
Expression heatmaps of *Fragaria* × *ananassa* JA signalling-related genes during fruit development and ripening. Expression heatmaps of *FaCOI1*, *FaMYC*s, *FaJAZ*s (A) and *FaNINJA*, *FaTPL*s, *FaHDA*s, *FaJAM*s (B). The Log-transformed values of relative expression levels based on RT-qPCR assays were used to perform heatmaps. The color scale represents relative expression levels with red and blue colors as high and low values, respectively. Black means no detection. The expression level of *FaGAPDH* was used as reference gene to normalize each reaction. The data was from three biological and three technical replicates. Developmental stages correspond to 0 (flowering, F), 10 (small green, SG), 17 (large green, LG), 20 (white, W), 21 (turning, T), 23 (50% red receptacle, 50%R) and 25 (100% red receptacle, 100%R) days after anthesis (DAA) in *F*. × *ananassa* cv. Aromas. JAZ, jasmonate ZIM-Domain.

To determine the expression dynamics of *FvJAZ* and *FvMYC* genes during fruit development and ripening, RT-qPCR assays were performed, and expression changes were represented by heatmaps (Fig 7A, S7 and S8 Tables). We included in the analysis the JA-Ile co-receptor COI1-encoding gene (*FaCOI1*), which showed two peaks in expression levels at F and T stages during fruit development and ripening (Fig 7A). Overall, *F*. × *ananassa* JAZ encoding genes (*FaJAZ*s) were downregulated from 0 to 25 DAA (F to R stages) (Fig 7A). Notably, the expression of *FaJAZ1*, *FaJAZ5* and *FaJAZ8*.*1* exhibited the highest reduction by 1043, 125 and 157-fold (p-value ≤ 0.05), respectively, from 0 to 25 DAA stages (F to R stages) (Fig 7A and S7 Table). These genes showed a similar expression pattern consisting in a reduction by 3.6, 2.3 and 3.4-fold (p-value ≤ 0.05) from 0 to 10 DAA (F to SG stages), a steady level from 10 to 21 DAA (SG to T stages), and then a reduction by 6.2, 4.3 and 6.8-fold (p-value ≤ 0.05), respectively, to 23 DAA (50%R stage) (Fig 7A and S7 Table). In contrast, the expression of *FaJAZ10* and *FaJAZ12* presented a similar reduction pattern from 0 to 25 DAA stages (F to R stages), being this reduction more pronounced for *FaJAZ10* with undetected levels at 23 and 25 DAA (50%R and R stages) (Fig 7A and S7 Table). Other *JAZ* genes (e.g., *FaJAZ4*s, *FaJAZ8*.*2 FaJAZ9*, *FaJAZ11*) also exhibited a higher expression at F stage and then lower constant levels during fruit development and ripening (Fig 7A). *FaMYC2* and *FaMYC2-like* showed an expression reduction during fruit development in a similar way to that observed for *FaJAZ*s (Fig 7A) although a greater expression decline was observed for *FaMYC2* than *FaMYC2-like* between 0 and 10 DAA (F and SG stages) (Fig 7A and S7 Table). Higher relative expression levels were observed for *FaMYC2* and *FaMYC2-like* in 0 and 10 DAA (F and SG stages) and a constant decrease was registered through fruit development and ripening (Fig 7A and S7 Table).

### Expression of other JA signalling-related genes during development and ripening in *F*. × *ananassa* fruit

To characterize temporal expression of additional components of the JA-related repressor machinery during *F*. × *ananassa* fruit development, we analyzed the expression levels of the *NINJA* adaptor-, *TPL*-, *HDA* co-repressors- and *JAM*-encoding genes (Fig 7B, S7 and S8 Tables). *FaNINJA* showing a higher level at 0 and 10 DAA (F and SG stages) a then progressively declined to 25 DAA (R stage) (Fig 7B and S7 Table). During *F*. × *ananassa* fruit development, different expression patterns were detected for *FaTPL1*, *FaTPL2*, *FaTPL3*, *FaTPL4*, *FaHDA6*.*1*, *FaHDA6*.*2*, *FaHDA19*.*1* and *FaHDA19*.*2* (Fig 7B and S7 Table). We also observed a constant reduction of *FaJAM1* and *FaJAM2* transcript accumulation through fruit development and ripening (Fig 7B) in accordance to the expression pattern of *FaMYC*s (Fig 7A). Overall, *F*. × *ananassa JAZ*s, *MYC2*, *MYC2-like* and other JA-signalling related genes are downregulated during fruit development and ripening.

### Expression of *FaJAZ1*, *FaJAZ8*.*1* and *FaMYC2* under JA treatment

**Fig 8:**
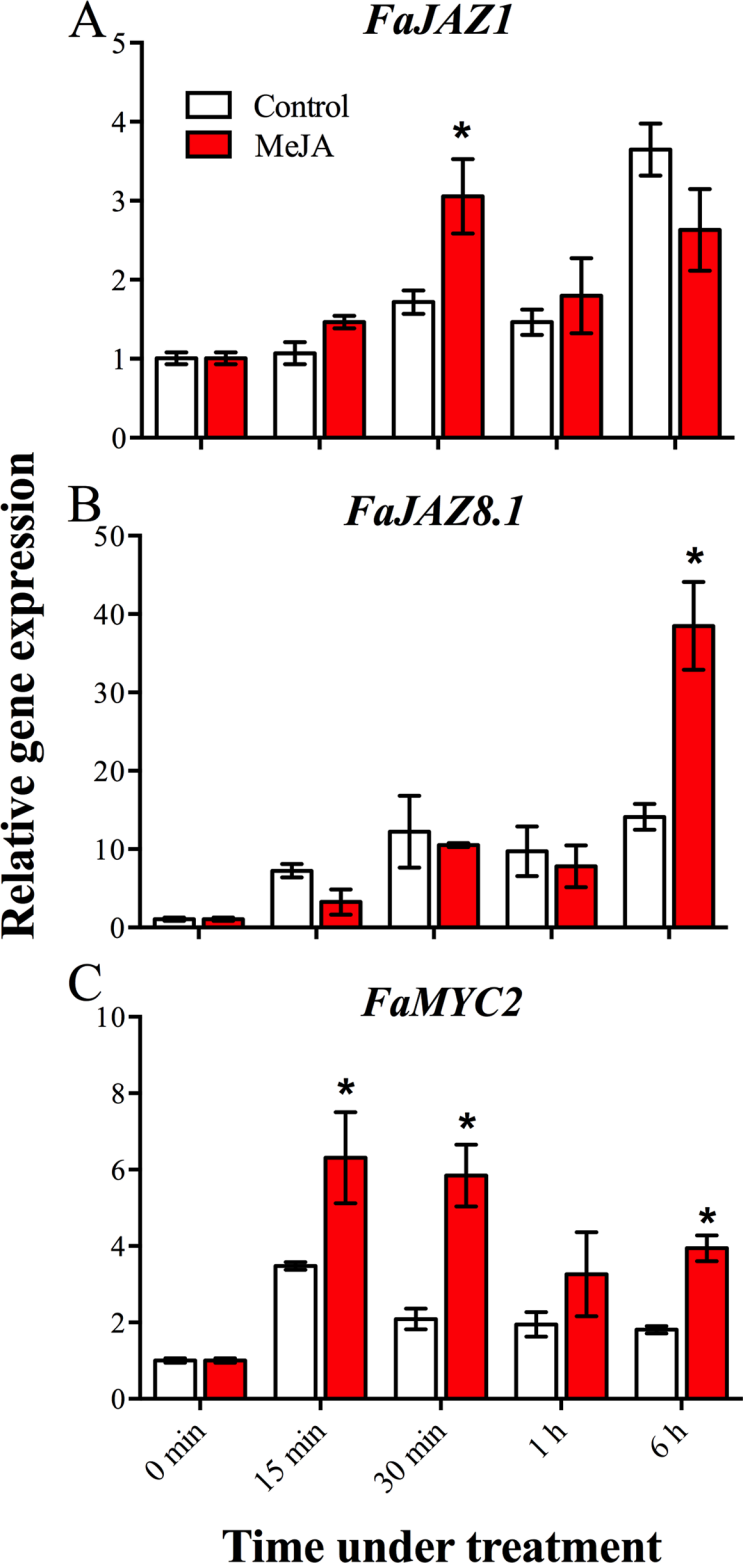
Expression of *FaJAZ1*, *FaJAZ8*.*1* and *FaMYC2* under MeJA treatment in *Fragaria* × *ananassa* fruit. Changes in relative expression of *FaJAZ1* (A), *FaJAZ8*.*1* (B) and *FaMYC2* (C) genes at 15 min, 30 min, 1 h and 6 h under 100 μM MeJA treatment. The expression level of *FaGAPDH* was used as reference gene to normalize each reaction. The data are from three biological and three technical replicates. Data were subjected to one-way ANOVA test, differences among means ± SE (n = 3) were determined using LSD test. Different letters indicate significant differences between developmental stages (p ≤ 0.05). JAZ, jasmonate ZIM-domain.

To gain insights into the expression response of *F*. × *ananassa JAZ* and *MYC* genes to JA treatment, we evaluated the expression profiles for *FaMYC2*, *FaJAZ1* and *FaJAZ8*.*1* in MeJA-treated W stage fruit (Fig 8 and S9 Table). We selected *FaJAZ1* and *FaJAZ8*.*1* because they showed the highest reduction in expression levels from 21 to 25 DAA (T and R stages) (Fig 7A), when *F*. × *ananassa* (cv. Aromas) starts the anthocyanin accumulation (S4 Fig).

*FaJAZ1* increased its expression 1.4-fold (p-value ≤ 0.05) respect to control after 30 min of 100 μM MeJA application in fruit (Fig 8A and S9 Table). *FaJAZ8*.*1* showed an upregulation of 2.7-fold (p-value ≤ 0.05) at 6 h after MeJA treatment with respect to a control (Fig 8B and S9 Table). On the other hand, *FaMYC2* exhibited a significant upregulation of 1.8, 2.8 and 2.2-fold (p-value ≤ 0.05) at 15 min, 30 min and 6 h after MeJA treatment, respectively (Fig 8C and S9 Table). These results demonstrate that *FaJAZ1*, *FaJAZ8*.*1* and *FaMYC2* are JA-responsive genes in *F*. × *ananassa* fruit. Moreover, *FaMYC2* responds earlier than *FaJAZ1* and *FaJAZ8*.*1* (Fig 8).

### Molecular characterization and expression of *PPD*s in strawberry

To gain insights into the existence of non-JAZ TIFY proteins in strawberry, we characterized inferred amino acid sequences containing conserved domains for TIFY and Jas. In this sense, we identified two *PEAPOD* (*PPD*) genes in *F*. *vesca* genome using AtPPD ortholog protein sequences as queries (S1 Table), named as *FvPPD1-1* and *FvPPD1-2* according to the gene nomenclature proposed for the Rosaceae [44]. We compared exon-intron structures between *F*. *vesca* and *A*. *thaliana PPD* genes and observed that *FvPPD1-1* and *FvPPD1-2* have an extra intron and longer introns in comparison to those of *A*. *thaliana* orthologs (Panel A in S5 Fig). FvPPDs proteins showed conserved position of PPD, TIFY and degenerated Jas domains respect to those present in AtPPD proteins (Panel B in S5 Fig) along with a high identity sequence as we observed from multiple sequence alignment analysis (Panel C in S5 Fig). Additionally, we constructed a phylogenetic tree that showed evolutionary relationships between FvPPDs and *A*. *thaliana*, *M*. x *domestica*, *V*. *vinifera* and *S*. *lycopersicum* orthologs (Panel D in S5 Fig). TIFY family was characterized in *O*. *sativa*, however PPDs were not reported [45]. FvPPD1-1 and FvPPD1-2 were clustered along with MdPPDs and VvPPD1 in group II (Panel D in S5 Fig). Finally, we determined the relative expression levels of *FaPPD1-1*, displaying a decreasing pattern during fruit development and ripening (Panel E in S5 Fig) as we observed for most of the JA signalling-related genes in strawberry.

## Discussion

### *FvJAZ*, *FvMYC2* and *FvMYC2-like* genes conserve synteny in *F*. *vesca* genome

JAZ and MYC along with COI1 co-receptor establish the core of JA signalling pathway [10,46]. Eleven to eighteen *JAZ* genes have been identified in the genome of higher plant species: thirteen JAZ protein members belonging to TIFY family in Arabidopsis [10,17], 15 members in rice [45], 14 members in wheat [47], 13 members in tomato [48], 18 members in apple and bamboo [49,50] and 11 members in grape [51]. We identified 12 non-redundant JAZ genes in *F*. *vesca* genome, *FvJAZ1*, *FvJAZ4-1*, *FvJAZ4-2*, *FvJAZ4-3*, *FvJAZ5*, *FvJAZ7*, *FvJAZ8*.*1*, *FvJAZ8*.*2*, *FvJAZ9*, *FvJAZ10*, *FvJAZ11* and *FvJAZ12* (Table 1). On the other hand, there are four MYC members involved in the JA signalling pathway in Arabidopsis: MYC2, MYC3, MYC4 and MYC5 [23–25]. Nevertheless, we only found two genes encoding for MYC TFs in *F*. *vesca* genome: *MYC2* and *MYC2-like* (Table 1). The number of *MYC2-like* genes is variable between species, for instance *M*. × *domestica* contains five *MYC2*–like TFs (S2 Table) [52,53], but others species such as *V*. *vinifera*, *Nicotiana tabacum*, *Nicotiana attenuata*, *Salvia miltiorrhiza*, and *S*. *lycopersicum* contain two *MYC2-like* TFs [51,54–56] like in *F*. *vesca* (Table 1 and S2 Table). In the case of apple, which belongs to Rosaceae family and is evolutionary related to strawberry, contains five MYC2*-like* encoding genes defined as *MdMYC2a* [52], *MdMYC2b*, *MdMYC2-like1*, *MdMYC2-like2* and *MdMYC2-like3* [53].

Tandem, segmental and whole duplication are key processes in the expansion of gene families [57,58] and genome comparisons provide information about roles and evolutionary relationships between genes [59]. Tandemly duplicated genes were considered as adjacent homologous in the same chromosome according to observed in rice and apple [45,50]. Specifically, gene duplications play an important role in expansion of the TIFY family [21], to which belongs JAZ subfamily as observed for *JAZ7*, *JAZ8* and *JAZ9*, *JAZ10* in *V*. *vinifera* and *S*. *lycopersicum* genomes, respectively [48,51], similar to that observed in apple *JAZ* genes [50]. These results indicate that *FvJAZ*, *AtJAZ*, *FvMYC2* and *AtMYC2* syntenic genes share likely a common ancestor and tandemly and segmental gene duplications were important for the expansion of JAZ subfamily [50]. In the case of *FvPPD* genes, they did not show syntenic regions within *A*. *thaliana* genome.

Exon-intron organization plays a role in diversification and evolution of gene families through gain/loss and insertion/deletions [58]. *FvJAZ* genes showed variable lengths and number of introns with their respective Arabidopsis orthologs (Fig 2A). These differences could be a consequence of rearrangements and fusions similar to that observed in apple *TIFY* gene family [50]. The presence of introns allows expanding the repertoire of some JAZ proteins, as reported for the different splice variants for AtJAZ10 with different stability in their encoded proteins and roles in JA responses [60]. In contrast to *FvJAZ* genes, *FvMYC2* and *FvMYC2-like* genes lack introns as Arabidopsis *MYC-like* orthologs (Fig 2B). In some cases, introns could have additional functions related to gene expression regulation [61], and this could be related with a key role in JAs responses [62–64], because their absence could be related with faster and efficient expression [65].

### FvJAZ proteins show conserved TIFY and Jas domains

To gain further insights in the primary protein structure and evolutionary relationships in JAZ and MYC protein families, multiple sequence alignment and phylogenetic analyses were performed (Figs 3–6, S2 and S3 Figs). FvJAZ proteins exhibited similar length and conserved structure according to that observed in Arabidopsis (Table 1) and previously reported in *V*. *vinifera* [51]. Analysis of the deduced amino acid sequence of *F*. *vesca* JAZs showed the conservation of the TIFY/ZIM domain that characterize this family (Figs 4A and 5A) [18,21] and a Jas domain (Figs 4B and 5B), which is specific of JAZ subfamily [10,20,21]. In *F*. *vesca*, the TIFY domain contains the highly conserved TIFY[F/Y]XG motif [18] (Figs 4A and 5A) as consensus sequence, however, some alternative sequences like TVFYXG and TIFFXG were found in FvJAZ10 and FvJAZ11, respectively (Fig 4A), as previously described for TIFY proteins in other species [21]. Jas domain maintained the conserved central amino acidic residues SLX_2_FLXKR[K/R]X[R/E] according to consensus sequence observed in *F*. *vesca* (Figs 4B and 5B) and similar to the previously reported SLX_2_FX_2_KRX_2_R sequence in Arabidopsis [20]. Besides, FvJAZ7, FvJAZ8.1 and FvJAZ8.2 displayed a variant Jas sequence (Fig 4B and S3 Fig) similar to that observed in Arabidopsis for JAZ7 and JAZ8 proteins [66]. Most of Arabidopsis JAZs contain a degron sequence at the Jas domain that is necessary for interaction with COI1 and JA-Ile [12], but only FvJAZ10 showed the canonical degron LPIAR(R/K) previously described for AtJAZ1, AtJAZ2, AtJAZ10 and AtJAZ12 proteins [66]. Other FvJAZ proteins displayed variations in degron sequences such as IPMQRK in FvJAZ1 (Fig 4B). Moreover, the degron sequence displayed the conserved residues R(K), and S and R (Fig 4B) interacting with COI1 [12] and MYC3 [67], respectively. Therefore, NLS located in C-terminal region [68] showed amino acidic residues conserved among JAZ proteins of *F*. *vesca* (Figs 4B and 5B). In some Arabidopsis JAZs, the N-terminal LxLxL type of EAR motif allows recruitment of TPL co-repressors to repress JA signalling pathway through a NINJA independent molecular mechanism [66]. In this regard, FvJAZ5 displayed the LxLxL type of EAR-motif at C-terminus, and FvJAZ7, FvJAZ8.1 and FvJAZ8.2 presented it at the N-terminus (S2 Fig) similar to that reported for AtJAZ5 and AtJAZ6, and AtJAZ7 and AtJAZ8 proteins, respectively (Fig 3) [66,69]. FvJAZ5 showed the lack of DLNEPT type of EAR-motif (Panel A in S2 Fig) in N-terminal region previously described in AtJAZ5 and AtJAZ6 proteins [66,69]. Phylogenetic analysis displayed evolutionary relationships between *F*. *vesca*, *A*. *thaliana*, *M*. × *domestica*, *V*. *vinifera*, *S*. *lycopersicum* and *O*. *sativa* JAZ proteins (Fig 6A). Protein grouping of FvJAZ proteins are related with high sequence identity respect to AtJAZ proteins (Fig 3A and S4 Table). The FvJAZ proteins clustered together with their Arabidopsis orthologs in a similar fashion than observed in several plant species [45,48,50,51], however, some phylogenetic trees contain lower bootstrap values [51]. At the same time, protein positions of FvJAZs in tree groups (Fig 6A) is in agreement with the identity and synteny analyses (Fig 1 and S1 Fig).

### FvMYC2 and FvMYC2-like proteins showed high conservation in JID and bHLH domains

In Arabidopsis, MYC2 [64], MYC3, MYC4 [23,70] and MYC5 [25] are master regulators of JA responses. We analyzed conservation domain in FvMYC2 and FvMYC2-like proteins and compared with AtMYC TFs (Fig 3B and S6 Table). The JID domain, which interacts with Jas domain to regulate JA responses [23,27], was present and conserved in FvMYC2 and FvMYC2-like proteins (Figs 3B, 4C and 5C). Both proteins exhibited conserved bHLH domains (Figs 3B, 4D and 5D), which bind to G-box *cis* elements in JA-response promoters [23–25]. The observed close evolutionary distances, the higher similarity in the identity matrix (S5 Table), and similar sequence lengths and domain positions (Fig 3B and S6 Table) suggest that FvMYC2 is the ortholog TF to AtMYC2. On the other hand, FvMYC2-like is phylogenetically closer to other MYC2-like TFs such as VvMYC2-like or MdMYC2-like TFs (Fig 6B). Furthermore, AtMYC3 and AtMYC4 were clustered as an independent group without ortholog sequences observed in *F*. *vesca* genome (Fig 6B) as previously reported in *N*. *attenuata* and *S*. *miltiorrhiza*, which MYC2 has been grouped in different clusters respect to MYC3 and MYC4 proteins [55,56].

### *FaMYC2*, *FaJAZ1* and *FaJAZ8*.*1* are downregulated during development and ripening in *F*. × *ananassa* fruit

We analyzed the expression profiles of *JAZ*s, *MYC2*, *MYC2-like* and other JA signalling-related genes during fruit development and ripening in *F*. × *ananassa*, the worldwide cultivated strawberry species. Most molecular studies on this species have been performed based on *F*. *vesca* reference genome [32] which is a subgenome of *F*. × *ananassa* [71]. Expression of the JA-Ile co-receptor *FaCOI1* (Fig 7A) was similar to that previously reported in *F*. × *ananassa* cv. Elsanta fruit [72]. In general, *FaJAZ* genes displayed a constant reduction pattern from flowering to ripe fruits, and some genes like *FaJAZ1*, *FaJAZ5* and *FaJAZ8*.*1* showed a pronounced reduction at ripe stages (Fig 7A), according to proanthocyanidins (PAs) reduction and opposite to anthocyanin accumulation during fruit development and ripening of cv. Aromas (S4 Fig). On the other hand, *FaJAZ10* was not detected in full ripe fruit (Fig 7A). Recently, similar expression profiles for *JAZ* genes were reported for a *JAZ* gene from large green to partial red fruit stages in *F*. × *ananassa* cv. Hongyan [31]. In this sense, Sánchez-Sevilla et al. also reported expression reduction for the most *JAZ*, named like *TIFY* genes, from RNAseq experiments in achene and receptacle during strawberry (*F*. × *ananassa* cv. Camarosa) fruit development and ripening (Panel A in S6 Fig and S10 Table) [73]. Moreover, *FaMYC2* and *FaMYC2-like* genes exhibiting an expression reduction from flowering to ripe stages, similar to that observed for *FaJAZ*s (Fig 7A) and to that previously reported both in achene and receptacle (Panel A in S6 Fig and S10 Table) [73]. *MYC2-like* genes have been reported in tomato and grape, although their expression patterns in fruit stages are unknown [54,74]. Globally, downregulation of *FaJAZ*s and *FaMYC2* genes (Fig 7A) matches with previously reported JA-Ile endogenous levels and expression of JA metabolism-related genes during *F*. × *ananassa* fruit development and ripening [7]. This suggests that JA metabolism and signalling are in coordination and JA pathway could have a key role in anthesis and physiological events like PAs biosynthesis that occur during early fruit development in strawberry (S4 Fig).

### Other JA-signalling genes are downregulated during development and ripening of *F*. × *ananassa* fruits

Other components of JA-signalling pathway like NINJA, TPLs and HDAs are key for JAs responses [10]. NINJA is necessary for transcriptional repression by interaction with JAZ proteins and the recruitment of TPL proteins [14,20]. *FaNINJA* showed the same expression pattern as *FaJAZ*, *FaMYC2* and *FaMYC2-like* genes during fruit development and ripening (Fig 7), like to previously observed for this gene in RNAseq assays (Panel B in S6 Fig and S10 Table) [73]. Otherwise, different patterns of expression were detected for *TPL* genes (Fig 7B) and these patterns were similar to those reported for *TPL* genes in *S*. *lycopersicum* fruit [75]. However, *TPL* gene expression seems to depend of the fruit tissue (Panel B in S6 Fig and S10 Table) [73]. Moreover, HDAs are recruited by TPLs during JAZ-mediated transcriptional repression [76], and *FaHDA6* and *FaHDA19* expression levels were similar to *FaTPL* genes (Fig 7B), suggesting a possible transcriptional coordination between both gene families. Finally, *FaJAM1* and *FaJAM2* exhibited a reduction expression during fruit development and ripening (Fig 7B), according to observed for *FaMYC2* and *FvMYC2-like* genes (Fig 7A) and expression levels previously reported for *F*. × *ananassa JAM1* and *JAM2* genes in receptacle by RNAseq assays (Panel B in S6 Fig and S10 Table) [73]. This fact is related with the antagonistic effect of JAMs on MYC2 TFs and the negative regulation of JA responses [28,29]. These results indicate that JA signalling pathway is turned off during development and ripening of *F*. × *ananassa* fruit according to the reduction of JA-Ile endogenous levels [7].

### *FaJAZ1*, *FaJAZ8*.*1* and *FaMYC2* are JA-responsive genes in *F*. × *ananassa* fruit

*JAZ* and *MYC2* genes are JA-responsive genes in Arabidopsis leaves [14]. We evaluated *FaJAZ1*, *FaJAZ8*.*1* and *FaMYC2* expression under 6 h of MeJA treatment. We selected *FaJAZ1* and *FaJAZ8*.*1* because they showed the highest reduction in expression levels from turning to ripe stages (Fig 7A), when *F*. × *ananassa* cv. Aromas starts the anthocyanin accumulation (S4 Fig). These genes also showed an expression pattern concomitant with the PAs accumulation pattern of developing fruit (S4 Fig). Moreover, we selected *FaJAZ1* and *FaJAZ8*.*1* since their deduced amino acid sequences are structurally different: FaJAZ1 contains a variation of the canonical degron sequence (IPMQRK) in contrast to FaJAZ8.1, which lacks canonical degron (Fig 4B) as well as AtJAZ8 [66]. *FaJAZ1* raised its expression levels after 30 min under MeJA treatment in fruit at white stage (Fig 8A), according to the previously observed with the ortholog genes in Arabidopsis leaves [14]. *FaJAZ8*.*1* displayed an increase of expression at 6 h after MeJA treatment, later than *FaJAZ1* (Fig 8B). On the other hand, *FaMYC2* showed higher expression levels at 15, 30 min and 6 h after MeJA application (Fig 8C), similar to observed for *AtMYC2* expression in Arabidopsis leaves [14]. In summary, *FaMYC2* responds earlier than *FaJAZ1* and *FaJAZ8*.*1* (Fig 8) suggesting a previous transcriptional activation according to the master role in JA responses regulation [10,11]. Furthermore, the early induction of *FaJAZ1*, *FaJAZ8*.*1* and *FaMYC2* genes could be related with JA-Ile accumulation under JA treatment, as previously reported [7].

## Conclusions

Overall, we identified and characterized 12 *JAZ* and two *MYC* genes in *F*. *vesca* genome, encoding for key components in the regulation of JA responses in plants [10,46]. Nevertheless, the number of *JAZ* and *MYC* genes could be higher to that reported in the present research, since *F*. x *ananassa* is an octoploid and hybrid species [71], similar to the findings reported for the triploid *M*. × *domestica* species [50]. Synteny analysis using Arabidopsis genome and exon-intron organization indicates that *FvJAZ* subfamily and *FvMYC2*-*like* genes have a common ancestor with Arabidopsis. Besides, protein sequences are highly conserved through domains and position into JAZ and MYC2-like proteins. Finally, we evaluated temporal expression pattern of key JA signalling components during development and ripening of *F*. × *ananassa* fruit, indicating that JA signalling pathway is downregulated along with PAs decrease accumulation during fruit development and ripening processes in agreement with previously reported temporal JA-Ile reduction [7]. In addition, we demonstrated that *FaMYC2*, *FaJAZ1* and *FaJAZ8*.*1* are JA-responsive genes in *F*. × *ananassa* fruit which could related with the activation of JA-Ile biosynthesis detected in MeJA-treated fruit recently reported [7].

The JA signalling pathway could trigger PAs biosynthesis at early stages of strawberry fruit development, which shows a similar reduction profile with JA pathway during development and ripening. Thus, the present research opens the gates to further studies to decipher specific JA-Ile roles and its signalling pathway-associated components during early development of strawberry and other non-climacteric fruits.

## Supporting information

S1 Fig Synteny analysis between *Arabidopsis thaliana* and *Malus* × *domestica*, *Solanum lycopersicum*, *Vitis vinifera* and *Oryza sativa JAZ* and *MYC* genes. Grey and blue horizontal lines indicate position of *JAZ* and *MYC-like* genes along chromosome, respectively. Orange, pink, purple and yellow lines indicate syntenic regions between *A*. *thaliana* and *M*. × *domestica*, *S*. *lycopersicum*, *V*. *vinifera* and *O*. *sativa JAZ* and *MYC-like* genes, respectively.
(TIF)

S2 Fig EAR motifs in *Fragaria vesca* (Fv) y *Arabidopsis thaliana* (At) JAZ proteins. DLNPT (A) and EAR LxLxL (B) motifs of AtJAZ5, AtJAZ6, FvJAZ5, and EAR LxLxL motif of AtJAZ7, AtJAZ8, FvJAZ7, FvJAZ8.1 and FvJAZ8.2 (C). Red and blue colors indicate higher and lower amino acidic residues conservation, respectively. EAR, ethylene-responsive element binding factor-associated amphiphilic repression domain; JAZ, jasmonate ZIM-domain.
(TIF)

S3 Fig Multiple sequences alignment of degenerate Jas domain in *Arabidopsis thaliana* (At) and *Fragaria vesca* (Fv) JAZ7 and JAZ8 proteins. Asterisks (*) indicate conserved residues involved for JAZ-MYC interaction (Zhang et al. 2015). Red and blue colors indicate higher and lower amino acidic residues conservation, respectively. JAZ, jasmonate ZIM-domain; NLS, nuclear localization signalling.
(TIF)

S4 Fig Proanthocyanidins (PAs) and anthocyanins contents during development and ripening of *Fragaria* × *ananassa* (cv. Aromas) fruit. Data are from three biological and three technical replicates. Developmental stages correspond to 0 (flowering, F), 10 (small green, SG), 17 (large green, LG), 20 (white, W), 21 (turning, T), 23 (50% red receptacle, 50%R) and 25 (100% red receptacle, 100%R) days after anthesis (DAA) in *F*. × *ananassa* cv. Aromas fruit. Data were analysed by one-way ANOVA test, and differences among means ± SE (n = 3) were determined using LSD test. Different letters indicate significant differences between developmental stages (p ≤ 0.05) for each gene.
(TIF)

S5 Fig Molecular characterization of PEAPOD (PPDs) protein subfamily in *Fragaria vesca* and *Fragaria* × *ananassa*. Exon-intron organization of *Arabidopsis thaliana* (At) and *F*. *vesca* (Fv) *PPD* genes (A). Yellow and red bars indicate UTR regions and exons, respectively. Black interrupted lines indicate introns. Domain structure of *A*. *thaliana* and *F*. *vesca* PPD proteins (B). The relative position of each domain within each protein are displayed in colors. Multiple alignment sequences of *A*. *thaliana* and *F*. *vesca* PPD proteins (C). Red and blue colors indicate higher and lower amino acidic residues conservation, respectively. Phylogenetic analysis between PPDs proteins of *A*. *thaliana*, *F*. *vesca*, *M*. × *domestica*, *V*. *vinifera* and *S*. *lycopersicum* (D). The phylogenetic analysis was performed using full-length JAZ and MYC protein sequences. Nodes with bootstrap values > 70% are labelled and bootstrap values ≥ 95% show highlight bootstrap. Relative gene expression of *F*. × *ananassa PPD1-1* during fruit development and ripening of *F*. × *ananassa* cv. Aromas (E). Developmental stages correspond to 0 (flowering, F), 10 (small green, SG), 17 (large green, LG), 20 (white, W), 21 (turning, T), 23 (50% red receptacle, 50%R), and 25 (100% red receptacle, R) days after anthesis (DAA). Data were analyzed by one-way ANOVA test, and differences among means ± SE (n = 3) were determined using LSD test. Different letters indicate significant differences between developmental stages (p ≤ 0.05).
(TIFF)

S6 Fig Expression patterns of JA signalling-related genes in achene and receptacle during development and ripening of *Fragaria* × *ananassa* (cv. Camarosa) fruit from FPKM values obtained from RNAseq experiments (Sanchez-Sevilla et al. 2017; see S10 Table). Gene expression patterns of *FaCOI1*, *FaMYC*s, *FaJAZ*s (A) and *FaNINJA*, *FaJAM*s, *FaTPL*s, *FaHDA*s (B). Expression data were extracted from accession numbers of *F*. *vesca* (Sanchez-Sevilla et al. 2017) and we renamed according to the present research gene nomenclature. *FaJAZ12* gene was not found in RNAseq experiments from Sanchez-Sevilla et al. (2017). Developmental stages correspond to GA (green achene), GR (green receptacle), RA (ripe achene), RR (ripe receptacle), TA (turning achene), TR (turning receptacle), WA (white achene), WR (white receptacle). COI1, coronatine insensitive 1; HDA, histone deacetylase; JAM, jasmonate-associated MYC2-like; JAZ, jasmonate-ZIM-domain; NINJA, novel interactor of JAZ; TPL, TOPLESS.
(TIFF)

S1 Table Predicted mRNA sequences of *Fragaria vesca* using JA signalling-related protein sequences of *Arabidopsis thaliana* as queries and Refseq-rna database of *F*. *vesca* as subject. COI1, coronatine insensitive 1; HDA, histone deacetylase; JAM, jasmonate-associated MYC2-like; JAZ, jasmonate-ZIM-domain; NINJA, novel interactor of JAZ; TPL, TOPLESS; PPD, PEAPOD.
(PDF)

S2 Table Genomic information of *JAZ* and *MYC* genes of *Arabidopsis thaliana*, *Fragaria vesca*, *Malus* × *domestica*, *Solanum lycopersicum*, *Vitis vinifera* and *Oryza sativa* used for synteny analysis. JAZ, jasmonate ZIM-domain.
(PDF)

S3 Table Primers sequences used for RT-qPCR analysis of the genes analyzed in this research. **The primers were designed from full-length cDNA sequences of *Fragaria vesca*. The primers for *COI1* and *GAPDH* genes were obtained from Preuss et al. (2014).** COI1, coronatine insensitive 1; GAPDH, glyceraldehyde-3-phospate dehydrogenase; HDA, histone deacetylase; JAM, jasmonate-associated MYC2-like; JAZ, jasmonate-ZIM-domain; NINJA, novel interactor of JAZ; TPL, TOPLESS; PPD, PEAPOD.
(PDF)

S4 Table Identity (%) between *Arabidopsis thaliana* and *Fragaria vesca* JAZ proteins obtained by multiple alignment. Bold numbers indicate the highest identity of *F*. *vesca* TIFY and JAZ proteins comparing to *Arabidopsis*. JAZ, jasmonate ZIM-domain.
(PDF)

S5 Table Identity (%) between MYCs transcription factors sequences of *Arabidopsis thaliana* and *Fragaria vesca* obtained by multiple alignment. Bold numbers indicate the highest identity of *F*. *vesca* MYCs comparing to *Arabidopsis*.
(PDF)

S6 Table Basic information of JAZ/TIFY and MYC proteins of Arabidopsis and *Fragaria vesca*. Arabidopsis and *F*. *vesca* TIFY, JAZ and MYCs proteins were obtained from GenPept database (NCBI).
(PDF)

S7 Table Relative expression of JA signalling-related genes during development and ripening of *Fragaria* × *ananassa* (cv. Aromas) fruit by RT-qPCR analysis. Developmental stages correspond to 0 (flowering, F), 10 (small green, SG), 17 (large green, LG), 20 (white, W), 21 (turning, T), 23 (50% red receptacle, 50%R), and 25 (100% red receptacle, R) days after anthesis (DAA). Data were analyzed by one-way ANOVA test, and differences among means ± SE (n = 3) were determined using LSD test. Different letters indicate significant differences between developmental stages (p ≤ 0.05) for each gene. nd = no detection. COI1, coronatine insensitive 1; HDA, histone deacetylases; JAM, jasmonate-associated MYC2-like; JAZ, jasmonate ZIM-domain; NINJA, novel interactor of JAZ; TPL, TOPLESS.
(PDF)

S8 Table Log_10_ transformed values used for heatmaps construction of JA signalling-related genes during development and ripening of *Fragaria* × *ananassa* (cv. Aromas) fruit. Developmental stages correspond to 0 (flowering, F), 10 (small green, SG), 17 (large green, LG), 20 (white, W), 21 (turning, T), 23 (50% red receptacle, 50%R), and 25 (100% red receptacle, R) days after anthesis (DAA). nd = no detection. COI1, coronatine insensitive 1; HDA, histone deacetylases; JAM, jasmonate-associated MYC2-like; JAZ, jasmonate ZIM-domain; NINJA, novel interactor of JAZ; TPL, TOPLESS.
(PDF)

S9 Table Relative expression of *FaMYC2*, *FaJAZ1* and *FaJAZ8*.*1* in MeJA-treated strawberry (*Fragaria* × *ananassa* cv. Aromas) fruit by RT-qPCR analysis. Expression levels were measured at 0, 15, 30 min, 1 and 6 h under MeJA treatment. At each treatment and time, three biological replicates were used for the different analysis. Data were analyzed by one-way ANOVA test, and differences among means ± SE (n = 3) were determined using LSD test. Asterisks (*) indicate significant differences between control and MeJA treatment (p ≤ 0.05) for each gene. JAZ, jasmonate ZIM-domain.
(PDF)

S10 Table Original FPKM values of JA signalling-related genes in achene and receptacle during development and ripening of *Fragaria* × *ananassa* (cv. Camarosa) fruit obtained from RNAseq experiments (Sanchez-Sevilla et al. 2017). Expression data were extracted from accession numbers of *F*. *vesca* (Sanchez-Sevilla et al. 2017) and we renamed according to the present research gene nomenclature. *FaJAZ12* gene was not found in RNAseq experiments from Sanchez-Sevilla et al. (2017). Developmental stages correspond to GA (green achene), GR (green receptacle), RA (ripe achene), RR (ripe receptacle), TA (turning achene), TR (turning receptacle), WA (white achene), WR (white receptacle). COI1, coronatine insensitive 1; HDA, histone deacetylases; JAM, jasmonate-associated MYC2-like; JAZ, jasmonate ZIM-domain; NINJA, novel interactor of JAZ; TPL, TOPLESS.
(PDF)
