# Correction: Jasmonate signalling pathway in strawberry: Genome-wide identification, molecular characterization and expression of *JAZ*s and *MYC*s during fruit development and ripening

**DOI:** 10.1371/journal.pone.0206559

**Published:** 2018-10-23

**Authors:** Adrián Garrido-Bigotes, Nicolás E. Figueroa, Pablo M. Figueroa, Carlos R. Figueroa

Several genes are labeled incorrectly in Fig 7. The authors have provided a corrected version here.

**Fig 7:**
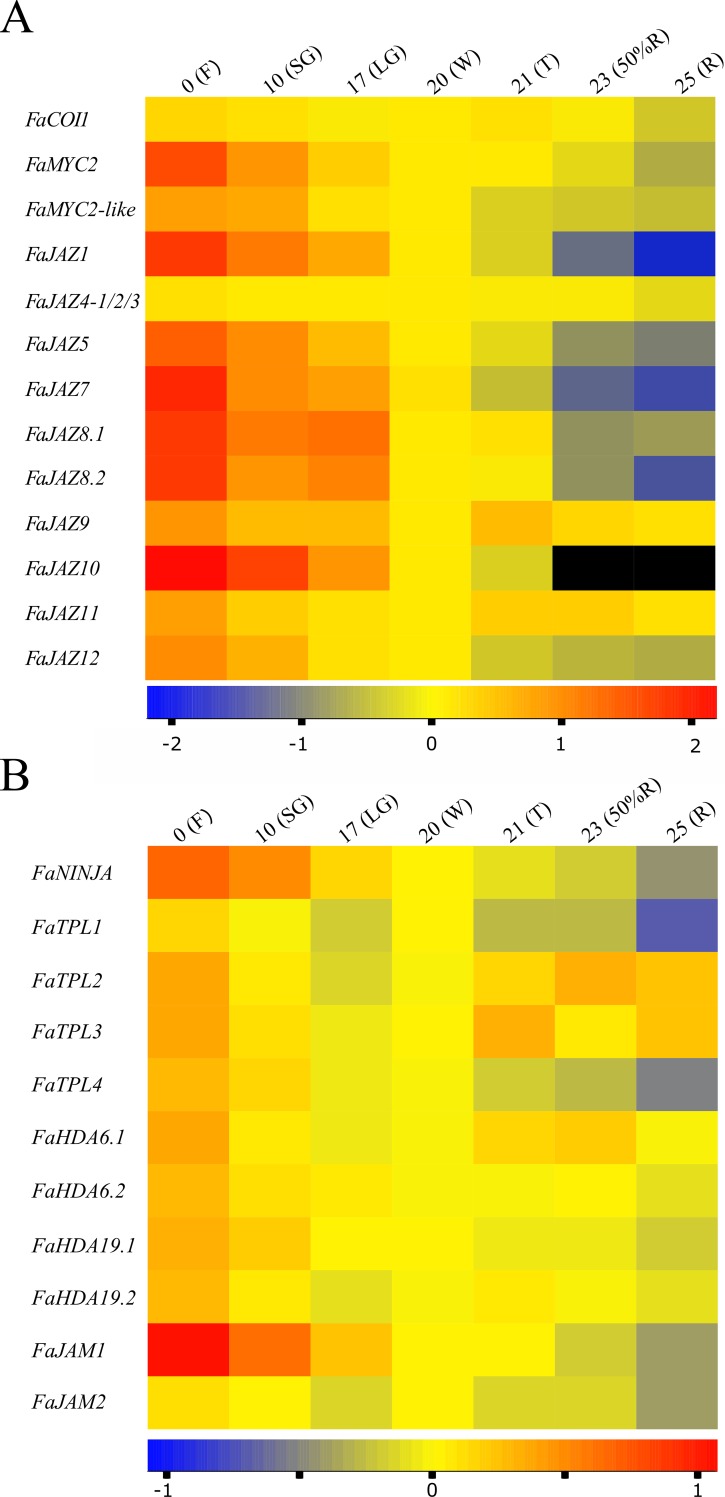
Expression heatmaps of *Fragaria × ananassa* JA signalling-related genes during fruit development and ripening. Expression heatmaps of *FaCOI1*, *FaMYCs*, *FaJAZs* (A) and *FaNINJA*, *FaTPLs*, *FaHDAs*, *FaJAMs* (B). The Log-transformed values of relative expression levels based on RT-qPCR assays were used to perform heatmaps. The color scale represents relative expression levels with red and blue colors as high and low values, respectively. Black means no detection. The expression level of *FaGAPDH* was used as reference gene to normalize each reaction. The data was from three biological and three technical replicates. Developmental stages correspond to 0 (flowering, F), 10 (small green, SG), 17 (large green, LG), 20 (white, W), 21 (turning, T), 23 (50% red receptacle, 50%R) and 25 (100% red receptacle, 100%R) days after anthesis (DAA) in *F*. *× ananassa* cv. Aromas. JAZ, jasmonate ZIM-Domain.
