# Supplementary figures and images for: Jasmonate signalling pathway in strawberry: Genome-wide identification, molecular characterization and expression of JAZs and MYCs during fruit development and ripening
Source: PLoS One. 2018 May 10;13(5):e0197118. doi: 10.1371/journal.pone.0197118 (PMC5944998; doi:10.1371/journal.pone.0197118)

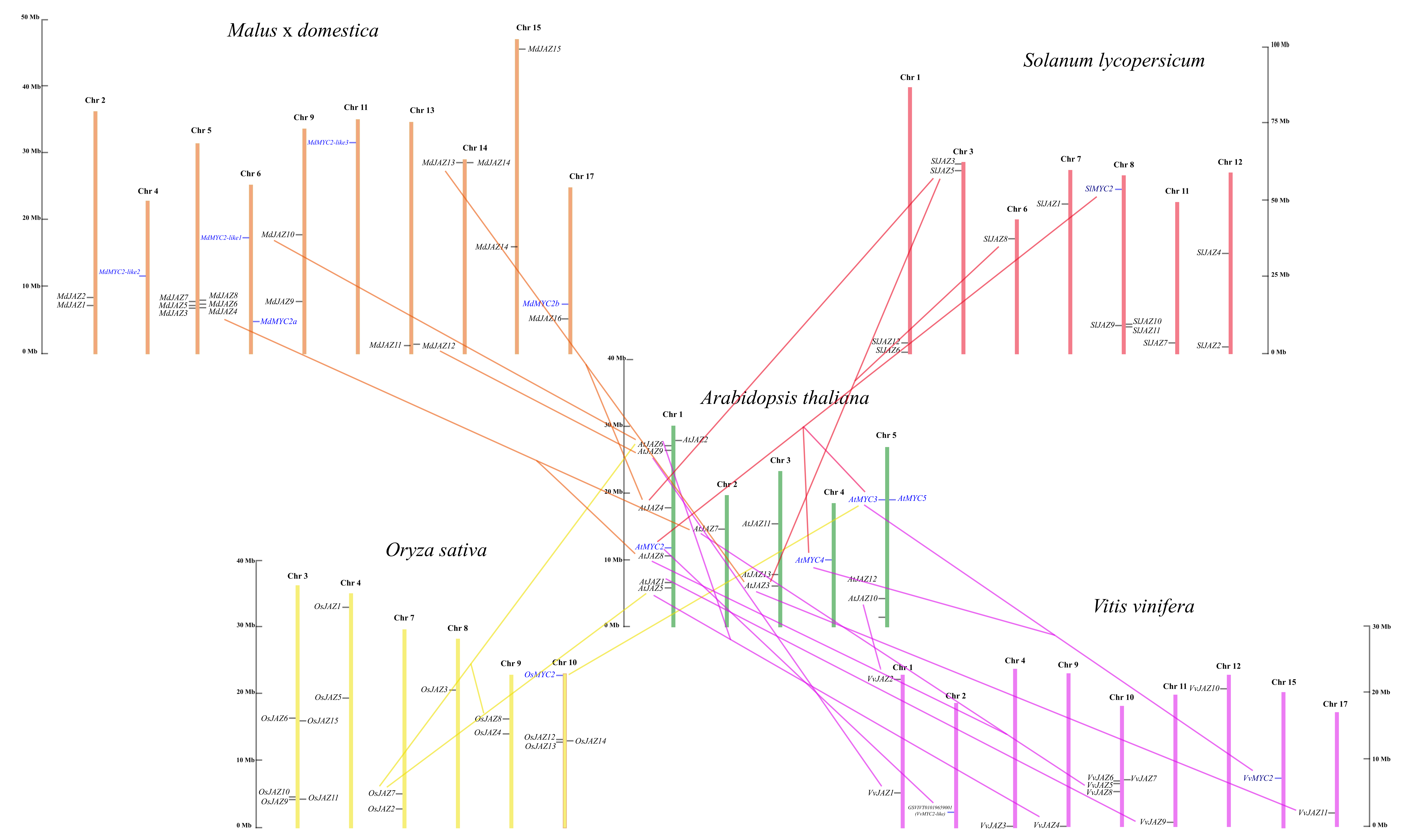

Supplement: S1 Fig — Grey and blue horizontal lines indicate position of JAZ and MYC-like genes along chromosome, respectively. Orange, pink, purple and yellow lines indicate syntenic regions between A. thaliana and M. × domestica, S. lycopersicum, V. vinifera and O. sativa JAZ and MYC-like genes, respectively. (TIF) [file pone.0197118.s001.tif]

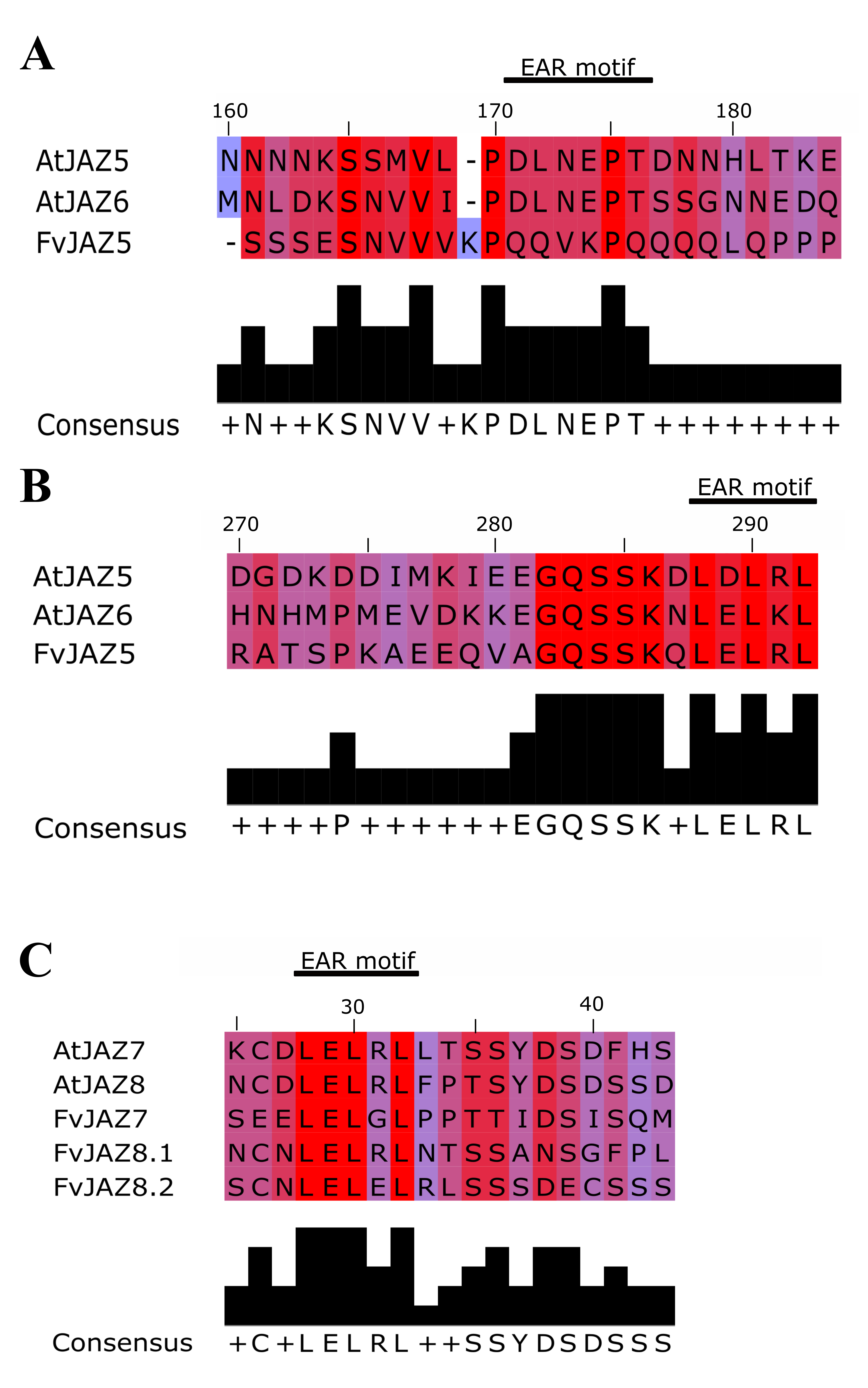

Supplement: S2 Fig — DLNPT (A) and EAR LxLxL (B) motifs of AtJAZ5, AtJAZ6, FvJAZ5, and EAR LxLxL motif of AtJAZ7, AtJAZ8, FvJAZ7, FvJAZ8.1 and FvJAZ8.2 (C). Red and blue colors indicate higher and lower amino acidic residues conservation, respectively. EAR, ethylene-responsive element binding factor-associated amphiphilic repression domain; JAZ, jasmonate ZIM-domain. (TIF) [file pone.0197118.s002.tif]

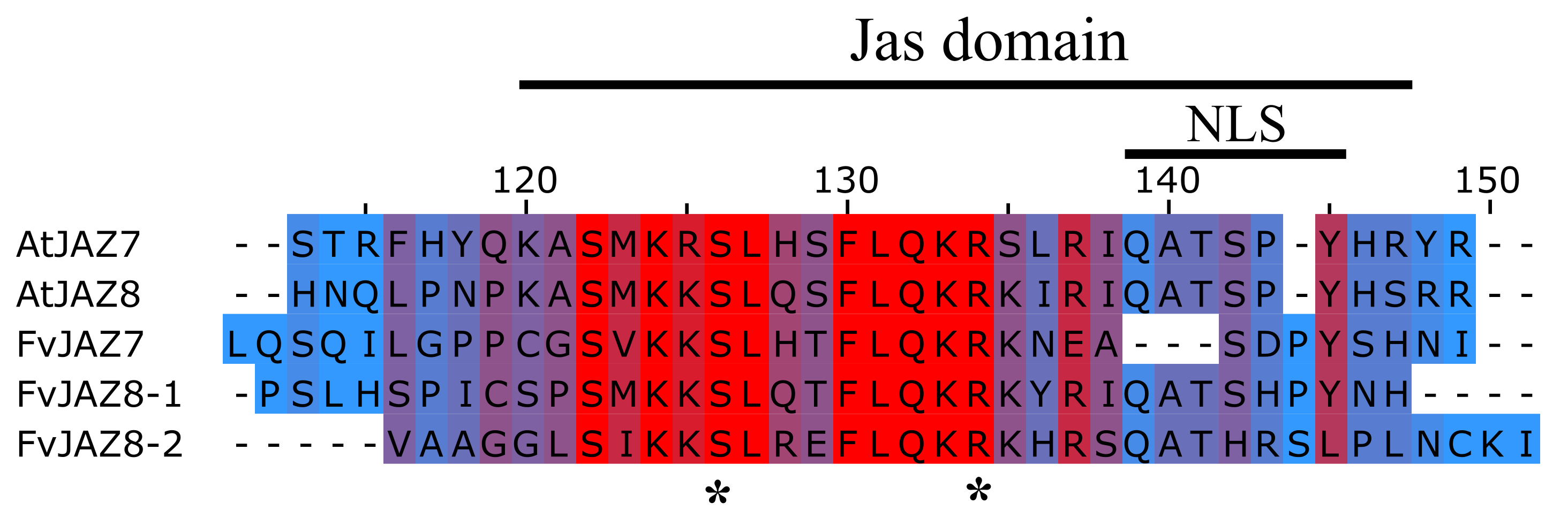

Supplement: S3 Fig — Asterisks (*) indicate conserved residues involved for JAZ-MYC interaction (Zhang et al. 2015). Red and blue colors indicate higher and lower amino acidic residues conservation, respectively. JAZ, jasmonate ZIM-domain; NLS, nuclear localization signalling. (TIF) [file pone.0197118.s003.tif]

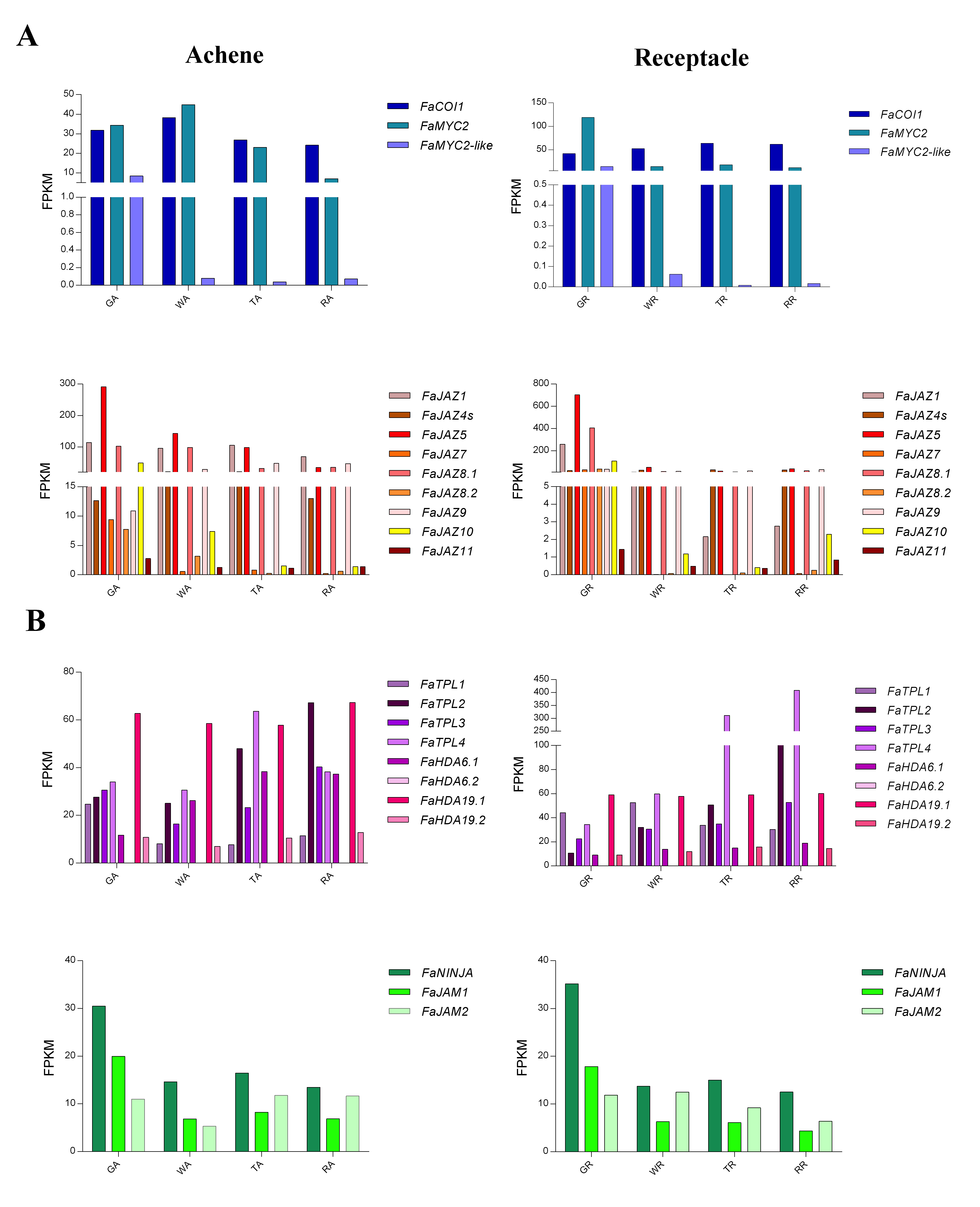

Supplement: S6 Fig — Gene expression patterns of FaCOI1, FaMYCs, FaJAZs (A) and FaNINJA, FaJAMs, FaTPLs, FaHDAs (B). Expression data were extracted from accession numbers of F. vesca (Sanchez-Sevilla et al. 2017) and we renamed according to the present research gene nomenclature. FaJAZ12 gene was not found in RNAseq experiments from Sanchez-Sevilla et al. (2017). Developmental stages correspond to GA (green achene), GR (green receptacle), RA (ripe achene), RR (ripe receptacle), TA (turning achene), TR (turning receptacle), WA (white achene), WR (white receptacle). COI1, coronatine insensitive 1; HDA, histone deacetylase; JAM, jasmonate-associated MYC2-like; JAZ, jasmonate-ZIM-domain; NINJA, novel interactor of JAZ; TPL, TOPLESS. (TIFF) [file pone.0197118.s006.tiff]
