# Supplementary material for: Jasmonate signalling pathway in strawberry: Genome-wide identification, molecular characterization and expression of JAZs and MYCs during fruit development and ripening
Source: PLoS One. 2018 May 10;13(5):e0197118. doi: 10.1371/journal.pone.0197118 (PMC5944998; doi:10.1371/journal.pone.0197118)
Supplement: S1 Table — COI1, coronatine insensitive 1; HDA, histone deacetylase; JAM, jasmonate-associated MYC2-like; JAZ, jasmonate-ZIM-domain; NINJA, novel interactor of JAZ; TPL, TOPLESS; PPD, PEAPOD. (PDF) [file pone.0197118.s007.pdf]

S1 Table. **Predicted mRNA sequences of *Fragaria vesca* using JA signalling-related protein sequences of *Arabidopsis thaliana* as queries and Refseq-rna database of *F. vesca* as subject.**

A. thaliana		tblastn				*Fragaria vesca*		
**Protein**	**Accession**	**Score**	**Query cover (%)**	**E-value**	**Identity (%)**	**Predicted mRNA**	**Accession**	**Name [a]**
AtJAZ1	NP_564075	127	99	2e-33	36	TIFY 10A (LOC101302102)	XM_004287607	*JAZ1/TIFY10a*
		107	60	2e-16	37	TIFY 11A-like (LOC101305492)	XM_004303663	*JAZ5/TFY11a*
AtJAZ2	NP_565096	122	93	2e-31	34	TIFY 10A (LOC101302102)	XM_004287607	*JAZ1/TIFY10a*
AtJAZ3	NP_566590	191	95	1e-54	42	TIFY 6B (LOC101303423)	XM_004299468	*JAZ9/TIFY6b*
		156	94	3e-42	38	TIFY 6B (LOC101298700), transcript variant X3	XM_011464880	*JAZ4-3/TIFY6b*
		146	94	2e-38	35	TIFY 6B (LOC101298700), transcript variant X2	XM_011464878	*JAZ4-2/TIFY6b*
		145	94	3e-38	35	TIFY 6B (LOC101298700), transcript variant X1	XM_004297401	*JAZ34-1/TIFY6b*
AtJAZ4	NP_001117450	138	99	1e-36	35	TIFY 6B (LOC101303423)	XM_004299468	*JAZ9/TIFY6b*
		127	99	1e-32	35	TIFY 6B (LOC101298700), transcript variant X3	XM_011464880	*JAZ4-3/TIFY6b*
		127	99	3e-30	34	TIFY 6B (LOC101298700), transcript variant X2	XM_011464878	*JAZ4-2/TIFY6b*
		120	99	4e-30	34	TIFY 6B (LOC101298700), transcript variant X1	XM_004297401	*JAZ4-1/TIFY6b*
AtJAZ5	NP_564019	75.5	75	4e-15	28	TIFY 10A-like (LOC101302102)	XM_004287607	*JAZ1/TIFY10a*
		68.6	77	7e-13	31	TIFY 11A-like (LOC101305492)	XM_004303663	*JAZ5/TFY11a*
AtJAZ6	NP_565043	74.7	78	8e-15	26	TIFY 10A-like (LOC101302102)	XM_004287607	*JAZ1/TIFY10a*
		100	68	9e-12	33	TIFY 11A-like (LOC101305492)	XM_004303663	*JAZ5/TFY11a*
AtJAZ7	NP_181007	68.9	85	3e-14	41	TIFY 5A-like (LOC101295112)	XM_004293578	*JAZ8.1/TIFY5a*
		42.4	56	6e-05	35	TIFY 5B (LOC105352369)	XM_011469361	*JAZ7/TIFY5b*
AtJAZ8	NP_564349	79.3	96	4e-18	43	TIFY 5A-like (LOC101295112)	XM_004293578	*JAZ8.1/TIFY5a*
AtJAZ9	NP_177227	94.4	98	3e-21	29	TIFY 6B (LOC101298700), transcript variant X3	XM_011464880	*JAZ4-3/TIFY6b*
		86.6	91	2e-19	31	TIFY 6B (LOC101303423)	XM_004299468	*JAZ9/TIFY6b*
		82.8	98	2e-17	28	TIFY 6B (LOC101298700), transcript variant X2	XM_011464878	*JAZ4-2/TIFY6b*
		82.4	98	3e-17	28	TIFY 6B (LOC101298700), transcript variant X1	XM_004297401	*JAZ4-1/TIFY6b*

AtJAZ10	NP_001154713	58.2	78	6e-10	32	TIFY 9-like (LOC101299545)	XM_004310081	*JAZ10/TIFY9*
AtJAZ11	NP_189930	137	78	1e-15	38	TIFY 3A-like (LOC105349490)	XM_011459279	*JAZ11/TIFY3a*
		122	75	4e-13	40	TIFY 3B (LOC101312185)	XM_004287641	*JAZ12/TIFY3b*
		53.5	41	7e-08	35	TIFY 9-like (LOC101299545)	XM_004310081	*JAZ10/TIFY9*
		47	77	8e-06	25	TIFY 6B (LOC101303423)	XM_011464880	*JAZ9/TIFY6b*
AtJAZ12	NP_197590	102	61	1e-25	48	TIFY 3B-like (LOC101312185)	XM_004287641	*JAZ12/TIFY3b*
		78.2	57	2e-17	41	TIFY 3A-like (LOC105349490)	XM_011459279	*JAZ11/TIFY3a*
		68.6	65	2e-13	30	TIFY 10A-like (LOC101302102)	XM_004287607	*JAZ1/TIFY10a*
		56.2	58	3e-09	36	TIFY 9-like (LOC101299545)	XM_004310081	*JAZ10/TIFY9*
AtJAZ13	NP_001078200	50.1	81	6e-08	32	TIFY 5B (LOC105352369)	XM_011469361	*JAZ7/TIFY5b*
AtNINJA	NP_849467	327	89	5e-95	45	Ninja-family protein mc410-like (LOC101314595)	XM_004287404	*NINJA*
AtTOPLESS	NP_849672	2004	100	0.0	81	TOPLESS-like (LOC101309271)	XM_004303220	*TPL1*
		1653	97	0.0	68	Topless-related protein 4-like (LOC101312083)	XM_004287239	*TPL2*
		1491	98	0.0	63	TOPLESS-like (LOC101312083)	XM_004307834	*TPL3*
		1391	99	0.0	60	Topless-related protein 2-like (LOC101296764)	XM_004296832	*TPL4*
AtHDA6	ACA97993	765	93	0.0	81	histone deacetylase 6-like (LOC101309835)	XM_004299084	*HDA6.1*
		651	87	0.0	71	histone deacetylase 6-like (LOC101314516)	XM_004289555	*HDA6.2*
AtHDA19	NP_195526	838	97	0.0	83	histone deacetylase 19-like (LOC101311924)	XM_004290052	*HDA19.1*
		531	79	3e-169	59	histone deacetylase 19-like (LOC101296668)	XM_004299996	*HDA19.2*
AtMYC2	NP_174541	584	100	0.0	49	transcription factor MYC2-like (LOC101308180)	XM_004300191	*MYC2*
		347	59	1e-54	53	transcription factor MYC2-like (LOC101299702)	XM_004306579	*MYC2-like*
AtMYC3	NP_19448	530	80	6e-83	59	transcription factor MYC2-like (LOC101308180)	XM_004300191	*MYC2*
		297	61	3e-45	47	transcription factor MYC2-like (LOC101299702)	XM_004306579	*MYC2-like*
AtMYC4	NP_193522	550	87	3e-90	62	transcription factor MYC2-like (LOC101308180)	XM_004300191	*MYC2*
		293	59	1e-44	47	transcription factor MYC2-like (LOC101299702)	XM_004306579	*MYC2-like*

AtMYC5	NP_199495	352	72	7e-51	56	transcription factor MYC2-like (LOC101308180)	XM_004300191	*MYC2*
		239	93	6e-70	34	transcription factor MYC2-like (LOC101299702)	XM_004306579	*MYC2-like*
AtJAM1	NP_566078	489	94	2e-163	48	transcription factor bHLH13-like (LOC101309083)	XM_004306609	*JAM1*
		330	96	1e-101	38	transcription factor bHLH3-like (LOC101302795)	XM_011459681	*JAM2*
AtJAM2	NP_171634	510	99	5e-171	47	transcription factor bHLH13-like (LOC101309083)	XM_004306609	*JAM1*
		327	71	5e-44	38	transcription factor bHLH3-like (LOC101302795)	XM_011459681	*JAM2*
AtJAM3	NP_193376	505	100	3e-170	53	transcription factor bHLH3-like (LOC101302795)	XM_011459681	*JAM2*
		340	93	3e-53	52	transcription factor bHLH13-like (LOC101309083)	XM_004306609	*JAM1*
AtPPD1	NP_567442	184	996	2e-53	40	TIFY 4B-like (LOC101296196), transcript variant X1	XM_004296526	*PPD1-1/TIFY4B*
		177	74	3e-51	43	TIFY 4B-like (LOC101296196), transcript variant X2	XM_011464153	*PPD1-2/TIFY4B*
AtPPD2	NP_193208	204	94	3e-61	42	TIFY 4B-like (LOC101296196), transcript variant X1	XM_004296526	*PPD1-1/TIFY4B*
		191	76	1e-56	44	TIFY 4B-like (LOC101296196), transcript variant X2	XM_011464153	*PPD1-2/TIFY4B*

COI1, coronatine insensitive 1; HDA, histone deacetylase; JAM, jasmonate-associated MYC2-like; JAZ, jasmonate-ZIM-domain; NINJA, novel interactor of JAZ; TPL, TOPLESS; PPD, PEAPOD.

[a] This refers to the gene names assigned in the present research.
