# Supplementary material for: Jasmonate signalling pathway in strawberry: Genome-wide identification, molecular characterization and expression of JAZs and MYCs during fruit development and ripening
Source: PLoS One. 2018 May 10;13(5):e0197118. doi: 10.1371/journal.pone.0197118 (PMC5944998; doi:10.1371/journal.pone.0197118)
Supplement: S2 Table — JAZ, jasmonate ZIM-domain. (PDF) [file pone.0197118.s008.pdf]

S2 Table. Genomic information of *JAZ* and *MYC* genes of *Arabidopsis thaliana*, *Fragaria vesca*, *Malus × domestica*, *Solanum lycopersicum*, *Vitis vinifera* and *Oryza sativa* used for synteny analysis.

Species	Gene	Accession	Database [a]	Chromosome	Start	End
Arabidopsis thaliana	*JAZ1*	AT1G19180	TAIR	1	6621777	6623620
	JAZ2	AT1G74950	TAIR	1	28148575	28150446
	JAZ3	AT3G17860	TAIR	3	6119707	6123044
	JAZ4	AT1G48500	TAIR	1	17931390	17934662
	JAZ5	AT1G17380	TAIR	1	5955155	5957511
	JAZ6	AT1G72450	TAIR	1	27273968	27276562
	JAZ7	AT2G34600	TAIR	2	14573030	14573952
	JAZ8	AT1G30135	TAIR	1	10596352	10597341
	JAZ9	AT1G70700	TAIR	1	26654529	26657350
	JAZ10	AT5G13220	TAIR	5	4218786	4221077
	JAZ11	AT3G43440	TAIR	3	15367546	15370157
	JAZ12	AT5G20900	TAIR	5	7090704	7092539
	JAZ13	AT3G22275	TAIR	3	7878807	7879810
	MYC2	AT1G32640	TAIR	1	11798119	11801407
	MYC3	AT5G46760	TAIR	5	18974022	18976590
	MYC4	AT4G17880	TAIR	4	9933326	9935685
	MYC5	AT5G46830	TAIR	5	19002564	19004384
Fragaria vesca	*JAZ1*	XM_004287607	NCBI	1	6964291	6965689
	JAZ4-1/2/3	XM_004297401/<br>XM_011464878/<br>XM_011464880	NCBI	4	19330995	19333777
	JAZ5	XM_004303663	NCBI	6	24774113	24774773
	JAZ7	XM_011469361	NCBI	6	27798266	27799489
	JAZ8.1	XM_004293578	NCBI	3	2612521	2613468
	JAZ8.2	XM_011461905	NCBI	3	2616234	2616765
	JAZ9	XM_004299468	NCBI	5	9961254	9964696
	JAZ10	XM_004310081	NCBI	1	1660803	1661807

	JAZ11	XM_011459279	NCBI	unknown	---	---
	JAZ12	XM_004287641	NCBI	1	7358182	7359713
	MYC2	XM_004306579	NCBI	5	21462454	21464502
	MYC2-like	XM_004300191	NCBI	7	2955350	2956825
Malus × domestica	*JAZ1*	MDP0000187921	GDR	2	7244327	7245983
	JAZ2	MDP0000301927	GDR	2	8372230	8374641
	JAZ3	MDP0000193833	GDR	5	6838732	6839413
	JAZ4	MDP0000135375	GDR	5	6838979	6839660
	JAZ5	MDP0000174042	GDR	5	6844271	6844929
	JAZ6	MDP0000718271	GDR	5	6853674	6854332
	JAZ7	MDP0000173534	GDR	5	7470093	7470870
	JAZ8	MDP0000173535	GDR	5	7471658	7472625
	JAZ9	MDP0000889413	GDR	9	7857488	7859513
	JAZ10	MDP0000565690	GDR	9	17833692	17834409
	JAZ11	MDP0000891920	GDR	13	1192483	1193511
	JAZ12	MDP0000452772	GDR	13	1265966	1268289
	JAZ13	MDP0000244580	GDR	14	28471434	28476544
	JAZ14	MDP0000243322	GDR	14	28498846	28504454
	JAZ15	MDP0000871409	GDR	15	15877982	15879552
	JAZ16	MDP0000285658	GDR	15	45582157	45585617
	JAZ17	MDP0000241358	GDR	17	5165874	5166628
	JAZ18	MDP0000757701	GDR	unknown	---	---
	MYC2a	MDP0000029168	GDR	6	4922683	4924743
	MYC2b	MDP0000136498	GDR	17	7285196	7287475
	MYC2-like1	MDP0000242554	GDR	6	20029017	20033121
	MYC2-like2	MDP0000900024	GDR	4	20793943	20795433
	MYC2-like3	MDP0000442310	GDR	11	33202192	33208027
Solanum lycopersicum	*JAZ1*	Solyc07g042170	Sol Genomics Network	7	55184447	55186127
	JAZ2	Solyc12g009220	Sol Genomics Network	12	2502141	2505029
	JAZ3	Solyc03g122190	Sol Genomics Network	3	70102121	70105461

	JAZ4	Solyc12g049400	Sol Genomics Network	12	36916955	36921569
	JAZ5	Solyc03g118540	Sol Genomics Network	3	67429700	67434430
	JAZ6	Solyc01g005440	Sol Genomics Network	1	308184	313133
	JAZ7	Solyc11g011030	Sol Genomics Network	11	4085260	4089578
	JAZ8	Solyc06g068930	Sol Genomics Network	6	42758254	42759870
	JAZ9	Solyc08g036640	Sol Genomics Network	8	10528948	10529900
	JAZ10	Solyc08g036620	Sol Genomics Network	8	10645788	10646615
	JAZ11	Solyc08g036660	Sol Genomics Network	8	10475721	10478023
	JAZ12	Solyc01g009740	Sol Genomics Network	1	4097401	4108551
	MYC2	Solyc08g076930	Sol Genomics Network	8	60869757	60872241
Vitis vinifera	*JAZ1*	GSVIVG01011679001	Vitis Genoscope	1	5284029	5288246
	JAZ2	GSVIVG01000967001	Vitis Genoscope	1	22328013	22331014
	JAZ3	GSVIVG01007188001	Vitis Genoscope	4	703	1712
	JAZ4	GSVIVG01016721001	Vitis Genoscope	9	647267	649385
	JAZ5	GSVIVG01021514001	Vitis Genoscope	10	6455988	6457349
	JAZ6	GSVIVG01021516001	Vitis Genoscope	10	6471500	6472777
	JAZ7	GSVIVG01021518001	Vitis Genoscope	10	6481575	6482572
	JAZ8	GSVIVG01021519001	Vitis Genoscope	10	5179406	5179432
	JAZ9	GSVIVG01015042001	Vitis Genoscope	11	683148	685564
	JAZ10	GSVIVG01023256001	Vitis Genoscope	12	20514823	20520056
	JAZ11	GSVIVG01008453001	Vitis Genoscope	17	1857303	1861568
	MYC2	GSVIVT00013156001	Vitis Genoscope	15	7426472	7428988
	MYC2-like	GSVIVT01019659001	Vitis Genoscope	2	2286157	2287242
Oryza sativa	*JAZ1*	Os04g55920	Rice Genome Anottation Project	2	33306461	33310232
	JAZ2	Os07g05830	Rice Genome Anottation Project	7	2804209	2801928
	JAZ3	Os08g33160	Rice Genome Anottation Project	8	20627940	20624903
	JAZ4	Os09g23660	Rice Genome Anottation Project	9	14060320	14055997
	JAZ5	Os04g32480	Rice Genome Anottation Project	4	19492574	19497154
	JAZ6	Os03g28940	Rice Genome Anottation Project	1	16410080	16407670
	JAZ7	Os07g42370	Rice Genome Anottation Project	7	25347985	25350251

	JAZ8	Os09g26780	Rice Genome Anottation Project	9	16275273	16273336
	JAZ9	Os03g08310	Rice Genome Anottation Project	1	4233042	4232001
	JAZ10	Os03g08330	Rice Genome Anottation Project	1	4248884	4249985
	JAZ11	Os03g08320	Rice Genome Anottation Project	1	4236724	4238274
	JAZ12	Os10g25290	Rice Genome Anottation Project	10	13070894	13069729
	JAZ13	Os10g25230	Rice Genome Anottation Project	10	13032006	13030986
	JAZ14	Os10g25250	Rice Genome Anottation Project	10	13046977	13042901
	JAZ15	Os03g27900	Rice Genome Anottation Project	1	16026190	16025189
	MYC2	Os10g42430	Rice Genome Anottation Project	10	22857123	22859886

JAZ, jasmonate ZIM-domain.

[a] Genome databases: *Arabidopsis thaliana* (TAIR10, http://www.arabidopsis.org/), *Fragaria vesca* (NCBI Genome, https://www.ncbi.nlm.nih.gov/genome/3314), *Malus* × *domestica* (GDR, https://www.rosaceae.org/), *Vitis vinifera* (Grape genome database, http://www.genoscope.cns.fr/externe/GenomeBrowser/Vitis/), *Solanum lycopersicum* (SolGenomics Network, https://www.solgenomics.net/) , *Oryza sativa* (Rice genome annotation project, http://rice.plantbiology.msu.edu/).
