# Supplementary material for: Jasmonate signalling pathway in strawberry: Genome-wide identification, molecular characterization and expression of JAZs and MYCs during fruit development and ripening
Source: PLoS One. 2018 May 10;13(5):e0197118. doi: 10.1371/journal.pone.0197118 (PMC5944998; doi:10.1371/journal.pone.0197118)
Supplement: S3 Table — The primers were designed from full-length cDNA sequences of Fragaria vesca. The primers for COI1 and GAPDH genes were obtained from Preuss et al. (2014). COI1, coronatine insensitive 1; GAPDH, glyceraldehyde-3-phospate dehydrogenase; HDA, histone deacetylase; JAM, jasmonate-associated MYC2-like; JAZ, jasmonate-ZIM-domain; NINJA, novel interactor of JAZ; TPL, TOPLESS; PPD, PEAPOD. (PDF) [file pone.0197118.s009.pdf]

**S3 Table. Primers sequences used for RT-qPCR analysis of the genes analyzed in this research. The primers were designed from full-length cDNA sequences of *Fragaria vesca*. The primers for *COI1* and *GAPDH* genes were obtained from Preuss et al. (2014).**

| Gene (CDS) | Forward (5'-3') | Reverse (5'-3') | Amplicon size (bp) |
|---|---|---|---|
| *COI1* | TGATCTTTTGGGGATGGCTCGCCC | TGAGCCGGATGCTCAACCACTACC | 117 |
| *JAZ1* | TGGGAGATCTGAACCTCGTC | TTCCTCGGTTTCTCCATCAC | 130 |
| *JAZ4-1/2/3* | AGAAGTGCTGGTGCACATTG | TGGGCATAAATCTGGAGGAC | 133 |
| *JAZ5* | CACCATGAACTTGCTCAACG | GAAAGGTCGCTGAAGACGAG | 126 |
| *JAZ7* | GGATGAGCAGACCAGACAGG | AAACATAAACCCGGCCATCG | 59 |
| *JAZ8.1* | GAGGAGGAACTGCAATTTGG | AAGAGGGAAGCCGGAATTAG | 64 |
| *JAZ8.2* | GTTGCAACCTGGAACTGGAAC | ATGAAGGGACTCAAGGGAGC | 71 |
| *JAZ9* | TCCACAGCTCAATTGCAAAC | ACCATCACCTGTGGAAGGAC | 51 |
| *JAZ10* | TTCCAGAAGTTCCTCGAACG | GATTTCCTGGCTGCAATCAC | 116 |
| *JAZ11* | GGTTGCAGAAAGGGCAAAGC | TTGCCAAAGGTATCCCTGCTC | 95 |
| *JAZ12* | GAAGCGTAGGGACAGATTGG | AACCGGAAGAAGCATCATTG | 104 |
| *MYC2* | AGGGGATCCTGTCGTTTACC | TTCGGGGTCCACAACTCTAC | 149 |
| *MYC2-like* | AGTCCATCCTCAACGACACC | AACCACTCTGCGTCGGATAC | 94 |
| *NINJA* | AAGCGATTTGTTGGATCTGG | ATTTGGGTCCACGCTACTTG | 84 |
| *TPL1* | TGCTTACTCGAGGCACATTG | GCTGCTTATTGGGGTGAGAG | 128 |
| *TPL2* | ATACTCTGTTTGCCCGCATC | TTCCATCAGTTGCTGTCGAG | 65 |
| *TPL3* | ATGATGCTCCTGGACTTTGG | TTCACCGTCTTTACCCGTTC | 89 |
| *TPL4* | CGAATCCTCTGTTCCGAGAG | AATGTCAGGATTTGGCCTTG | 128 |
| *HDA6.1* | GTTTGGCGATTTCTTTCCAG | TCCGAAAATTCTCGTCATCC | 116 |
| *HDA6.2* | ATGGAGCTGAATCGGGTTGG | GCAGTTAGTGTCAGGGGTGG | 101 |
| *HDA19.1* | CTACCTTCAGGACCAGACGC | TGGTTGAGGCCGTAGTTGAC | 149 |
| *HDA19.2* | GGACAAGCAGCATTTGTTCTC | ACCTACGTGCAGCATCTATCG | 128 |
| *JAM1* | GCTTTACGAGCTGTTGTGCC | TTGAAGTGCCTCCTGCACTC | 139 |
| *JAM2* | GACAATTGGGTGTGCAGCAG | TCCATCGCCCCAAATCAAGG | 122 |
| *PPD1-1* | CCTGAGGGTCAGGCAAGTAG | CTCCAAGGTAGAAGTTGGTCCC | 110 |
| *GAPDH* | TTCATCACTACTGCCACCCAGAAGACTG | AGCAGGCAGAACCTTTCCGACAG | 132 |

COI1, coronatine insensitive 1; GAPDH, glyceraldehyde-3-phospate dehydrogenase; HDA, histone deacetylase; JAM, jasmonate-associated MYC2-like; JAZ, jasmonate-ZIM-domain; NINJA, novel interactor of JAZ; TPL, TOPLESS; PPD, PEAPOD.
