# Supplementary material for: Jasmonate signalling pathway in strawberry: Genome-wide identification, molecular characterization and expression of JAZs and MYCs during fruit development and ripening
Source: PLoS One. 2018 May 10;13(5):e0197118. doi: 10.1371/journal.pone.0197118 (PMC5944998; doi:10.1371/journal.pone.0197118)
Supplement: S4 Table — Bold numbers indicate the highest identity of F. vesca TIFY and JAZ proteins comparing to Arabidopsis. JAZ, jasmonate ZIM-domain. (PDF) [file pone.0197118.s010.pdf]

S4 Table. **Identity (%) between *Arabidopsis thaliana* and *Fragaria vesca* JAZ proteins obtained by multiple alignment.**

	Fragaria vesca											
Arabidopsis thaliana	JAZ1	JAZ4-1	JAZ4-2	JAZ4-3	JAZ5	JAZ7	JAZ8.1	JAZ8.2	JAZ9	JAZ10	JAZ11	JAZ12
JAZ1	**40.08**	22.41	22.94	23.19	36.46	21.43	25.23	23.14	22.77	26.87	26.81	29.45
JAZ2	38.17	23.93	24.03	24.40	40.70	23.21	25.00	21.14	21.78	26.72	24.26	29.93
JAZ3	24.18	**39.12**	**38.94**	**39.82**	29.79	19.64	26.96	23.62	**45.92**	32.62	29.19	26.32
JAZ4	23.96	37.59	37.36	38.58	35.24	23.91	30.53	25.49	41.29	**36.13**	**32.71**	26.36
JAZ5	29.78	21.15	21.26	22.40	**43.86**	21.36	23.30	21.43	22.07	22.73	26.00	21.94
JAZ6	28.82	20.85	20.95	22.34	43.71	18.75	20.54	23.14	20.93	22.14	22.73	24.68
JAZ7	20.63	19.67	19.67	19.67	27.84	30.84	**47.01**	36.29	22.61	22.76	23.00	25.93
JAZ8	23.97	22.88	22.88	22.88	26.37	**36.79**	42.74	**36.89**	20.18	24.04	19.19	25.47
JAZ9	24.40	38.37	38.37	39.27	29.91	22.22	25.23	20.83	35.88	27.56	27.54	23.61
JAZ10	16.53	20.15	20.15	20.15	22.62	18.63	19.05	19.80	20.33	31.47	21.98	23.71
JAZ11	20.79	20.00	19.65	20.38	20.90	15.74	21.70	17.24	24.54	22.83	27.07	33.33
JAZ12	23.03	20.11	20.22	22.42	23.08	19.35	23.91	17.92	25.29	30.56	32.52	**40.36**
JAZ13	17.43	16.53	16.53	16.53	18.00	27.45	30.19	27.93	20.87	18.42	17.12	16.36

Bold numbers indicate the highest identity of *F. vesca* TIFY and JAZ proteins comparing to *Arabidopsis*.
JAZ, jasmonate ZIM-domain.
