# Supplementary material for: Jasmonate signalling pathway in strawberry: Genome-wide identification, molecular characterization and expression of JAZs and MYCs during fruit development and ripening
Source: PLoS One. 2018 May 10;13(5):e0197118. doi: 10.1371/journal.pone.0197118 (PMC5944998; doi:10.1371/journal.pone.0197118)
Supplement: S5 Table — Bold numbers indicate the highest identity of F. vesca MYCs comparing to Arabidopsis. (PDF) [file pone.0197118.s011.pdf]

S5 Table. **Identity (%) between MYCs transcription factors sequences of *Arabidopsis thaliana* and *Fragaria vesca* obtained by multiple alignment.**

		Fragaria vesca	
		MYC2	MYC2-like
Arabidopsis thaliana	MYC2	**58.51**	**41.54**
	MYC3	54.16	38.88
	MYC4	58.27	38.92
	MYC5	45.56	38.69

Bold numbers indicate the highest identity of *F. vesca* MYCs comparing to *Arabidopsis*.
