# Supplementary material for: Jasmonate signalling pathway in strawberry: Genome-wide identification, molecular characterization and expression of JAZs and MYCs during fruit development and ripening
Source: PLoS One. 2018 May 10;13(5):e0197118. doi: 10.1371/journal.pone.0197118 (PMC5944998; doi:10.1371/journal.pone.0197118)
Supplement: S6 Table — Arabidopsis and F. vesca TIFY, JAZ and MYCs proteins were obtained from GenPept database (NCBI). (PDF) [file pone.0197118.s012.pdf]

S6 Table. Basic information of JAZ/TIFY and MYC proteins of Arabidopsis and *Fragaria vesca*.

**Arabidopsis**				***Fragaria vesca***			
**Protein**	**Accession**	**Length (aa)**	**EAR motif**	**Protein [a]**	**Accession**	**Length (aa)**	**EAR motif**
JAZ1/TIFY10a	NP_564075	253		JAZ1/TIFY10a	XP_004287655	302	
JAZ2/TIFY10b	NP_565096	249		JAZ4.1/TIFY6b_X1	XP_004297449	385	
JAZ3/TIFY6b	NP_566590	352		JAZ4.2/TIFY6b_X2	XP_011463180	384	
JAZ4/TIFY6a	NP_001117450	310		JAZ4.3/TIFY6b_X3	XP_011463182	360	
JAZ5/TIFY11a	NP_564019	274	DLNEPT<br>LDLRL	JAZ5/TIFY11a	XP_004303711	185	LELRL
JAZ6/TIFY11b	NP_565043	269	DLNEPT<br>LELKL	JAZ7/TIFY5b	XP_011467663	123	LELGL
JAZ7/TIFY5b	NP_181007	148	LELRL	JAZ8.1/TIFY5a	XP_004293626	130	LELRL
JAZ8/TIFY5a	NP_564349	131	LELRL	JAZ8.2/TIFY5b	XP_011460207	139	LELRL
JAZ9/TIFY7	NP_177227	267		JAZ9/TIFY6b	XP_004299516	371	
JAZ10/TIFY9	NP_001154713	197		JAZ10/TIFY9	XP_004310129	191	
JAZ11/TIFY3a	NP_189930	238		JAZ11/TIFY3a	XP_011457581	186	
JAZ12/TIFY3b	NP_197590	187		JAZ12/TIFY3b	XP_011461243	202	
JAZ13	NP_001078200	125		MYC2	XP_004300239	682	
MYC2	NP_174541	623		MYC2-like	XP_004306627	491	
MYC3	NP_199488	592					
MYC4	NP_193522	589					
MYC5	NP_199495	511					

Arabidopsis and *F. vesca* TIFY, JAZ and MYCs proteins were obtained from GenPept database (NCBI).

[a] In GenPept database (NCBI) *Fragaria* JAZ proteins are named as TIFY proteins.
