# Supplementary material for: Jasmonate signalling pathway in strawberry: Genome-wide identification, molecular characterization and expression of JAZs and MYCs during fruit development and ripening
Source: PLoS One. 2018 May 10;13(5):e0197118. doi: 10.1371/journal.pone.0197118 (PMC5944998; doi:10.1371/journal.pone.0197118)
Supplement: S7 Table — Developmental stages correspond to 0 (flowering, F), 10 (small green, SG), 17 (large green, LG), 20 (white, W), 21 (turning, T), 23 (50% red receptacle, 50%R), and 25 (100% red receptacle, R) days after anthesis (DAA). Data were analyzed by one-way ANOVA test, and differences among means ± SE (n = 3) were determined using LSD test. Different letters indicate significant differences between developmental stages (p ≤ 0.05) for each gene. nd = no detection. COI1, coronatine insensitive 1; HDA, histone deacetylases; JAM, jasmonate-associated MYC2-like; JAZ, jasmonate ZIM-domain; NINJA, novel interactor of JAZ; TPL, TOPLESS. (PDF) [file pone.0197118.s013.pdf]

S7 Table. Relative expression of JA signalling-related genes during development and ripening of *Fragaria × ananassa* (cv. Aromas) fruit by RT-qPCR analysis.

Gene	Days after anthesis (developmental stages)						
	**0 (F)**	**10 (SG)**	**17 (LG)**	**20 (W)**	**21 (T)**	**23 (50%R)**	**25 (R)**
FaCOI1	1.62 ± 0.24 a	1.2 ± 0.18 ab	0.90 ± 0.08 bc	1.01 ± 0.07 bc	1.25 ± 0.31 ab	0.92 ± 0.15 bc	0.47 ± 0.19 c
FaMYC2	19.07 ± 2.37 a	5.62 ± 2.40 b	1.97 ± 1.10 b	1.15 ± 0.43 c	1.14 ± 0.27 c	0.69 ± 0.26 c	0.29 + 0.11 c
FaMYC2-like	4.16 ± 0.83 a	3.99 ± 1.20 a	1.40 ± 0.22 b	1.00 ± 0.06 b	0.51 ± 0.04 b	0.49 ± 0.07 b	0.37 ± 0.07 b
FaJAZ1	31.28 ± 1.42 a	8.72 ± 2.85 b	3.86 ± 1.95 bc	1.08 ± 0.32 cd	0.56 ± 0.18 cd	0.09 ± 0.02 d	0.03 ± 0.01 d
FaJAZ4-1/2/3	1.28 ± 0.06 a	1.10 ± 0.09 ab	1.02 ± 0.15 ab	1.01 ± 0.1 ab	0.94 ± 0.02 b	0.95 ± 0.11 b	0.61 ± 0.15 c
FaJAZ5	14.95 ± 1.19 a	6.49 ± 2.17 b	2.72 ± 1.15 c	1.15 ± 0.46 c	0.69 ± 0.13 c	0.16 ± 0.01 d	0.12 ± 0.01 d
FaJAZ7	43.08 ± 2.92 a	6.45 ± 3.90 a	4.14 ± 2.80 b	1.23 ± 0.49 b	0.41 ± 0.13 b	0.08 ± 0.01 b	0.04 ± 0.02 b
FaJAZ8.1	29.80 ± 4.00 a	8.88 ± 2.16 bc	10.72 ± 5.45 b	1.17 ± 0.49 cd	1.23 ± 0.34 cd	0.18 ± 0.05 d	0.19 ± 0.09 d
FaJAZ8.2	29.62 ± 8.63 a	5.56 ± 1.19 a	6.90 ± 3.07 b	1.05 ± 0.24 b	0.91 ± 0.21 b	0.16 ± 0.05 b	0.06 ± 0.01 b
FaJAZ9	5.51 ± 1.52 a	2.79 ± 0.37 b	2.53 ± 0.83 b	1.01 ± 0.12 b	2.67 ± 0.54 b	1.48 ± 0.18 b	1.39 ± 0.13 b
FaJAZ10	149.38 ± 5.75 a	46.62 ± 19.76 b	9.45± 7.85 c	1.03 ± 0.60 c	1.12 ± 0.41 c	nd	nd
FaJAZ11	3.31 ± 0.68 a	1.38 ± 0.29 b	0.87 ± 0.09 c	0.84 ± 0.01 c	1.41 ± 0.37 b	1.28 ± 0.29 b	0.94 ± 0.37 b
FaJAZ12	5.76 ± 0.20 a	3.34 ± 0.23 b	1.39 ± 0.15 c	1.01 ± 0.11 c	0.46 ± 0.07 d	0.34 ± 0.04 d	0.27 ± 0.01 d
FaNINJA	4.70 ± 1.15 a	3.17 ± 0.78 a	1.44 ± 0.43 b	1.03 ± 0.19 b	0.76 ± 0.01 b	0.62 ± 0.03 b	0.36 ± 0.04 b
FaTPL1	1.38 ± 0.04 a	0.92 ± 0.04 b	0.65 ± 0.17 c	1.02 ± 0.13 b	0.51 ± 0.03 c	0.53 ± 0.08 c	0.21 ± 0.03 d
FaTPL2	2.34 ± 0.10 a	1.16 ± 0.17 cde	0.75 ± 0.17 e	1.01 ± 0.08 de	1.37 ± 0.10 cd	2.07 ± 0.32 ab	1.65 ± 0.19 bc
FaTPL3	2.18 ± 0.05 a	1.26 ± 0.12 b	0.85 ± 0.19 b	1.02 ± 0.13 b	2.11 ± 0.24 a	1.12 ± 0.16 b	1.76 ± 0.13 a
FaTPL4	1.92 ± 0.06 a	1.44 ± 0.15 b	0.85 ± 0.15 cd	1.00 ± 0.06 c	0.63 ± 0.07 de	0.55 ± 0.05 ef	0.30 ± 0.01 f
FaHDA6.1	2.26 ± 0.17 a	1.11 ± 0.08 bc	0.86 ± 0.06 c	1.00 ± 0.02 bc	1.36 ± 0.35 bc	1.56 ± 0.41 b	1.01 ± 0.13 bc
FaHDA6.2	1.80 ± 0.09 a	1.30 ± 0.11 b	1.13 ± 0.09 bc	1.00 ± 0.04 c	0.94 ± 0.04 cd	1.07 ± 0.06 bc	0.75 ± 0.08 d
FaHDA19.1	2.14 ± 0.14 a	1.55 ± 0.18 b	1.09 ± 0.30 bc	1.02 ± 0.13 c	0.89 ± 0.20 b	0.85 ± 0.05 b	0.65 ± 0.04 c
FaHDA19.2	1.89 ± 0.09 a	1.15 ± 0.09 b	0.78 ± 0.11 c	1.01 ± 0.08 bc	1.15 ± 0.13 b	0.99 ± 0.07 bc	0.80 ± 0.18 c
FaJAM1	11.46 ± 2.42 a	4.14 ± 1.33 b	1.74 ± 0.55 bc	1.04 ± 0.20 bc	1.07 ± 0.08 bc	0.67 ± 0.04 c	0.39 ± 0.05 c
FaJAM2	1.33 ± 0.03 a	1.06 ± 0.12 a	0.70 ± 0.18 bcd	1.02 ± 0.14 ab	0.73 ± 0.07 bc	0.69 ± 0.05 cd	0.39 ± 0.07 d

Developmental stages correspond to 0 (flowering, F), 10 (small green, SG), 17 (large green, LG), 20 (white, W), 21 (turning, T), 23 (50% red receptacle, 50%R), and 25 (100% red receptacle, R) days after anthesis (DAA). Data were analyzed by one-way ANOVA test, and differences among means ± SE (n = 3) were determined using LSD test. Different letters indicate significant differences between developmental stages ($p \leq 0.05$) for each gene. nd = no detection. COI1, coronatine insensitive 1; HDA, histone deacetylases; JAM, jasmonate-associated MYC2-like; JAZ, jasmonate ZIM-domain; NINJA, novel interactor of JAZ; TPL, TOPLESS.
