# Supplementary material for: Jasmonate signalling pathway in strawberry: Genome-wide identification, molecular characterization and expression of JAZs and MYCs during fruit development and ripening
Source: PLoS One. 2018 May 10;13(5):e0197118. doi: 10.1371/journal.pone.0197118 (PMC5944998; doi:10.1371/journal.pone.0197118)
Supplement: S8 Table — Developmental stages correspond to 0 (flowering, F), 10 (small green, SG), 17 (large green, LG), 20 (white, W), 21 (turning, T), 23 (50% red receptacle, 50%R), and 25 (100% red receptacle, R) days after anthesis (DAA). nd = no detection. COI1, coronatine insensitive 1; HDA, histone deacetylases; JAM, jasmonate-associated MYC2-like; JAZ, jasmonate ZIM-domain; NINJA, novel interactor of JAZ; TPL, TOPLESS. (PDF) [file pone.0197118.s014.pdf]

S8 Table. Log_{10} transformed values used for heatmaps construction of JA signalling-related genes during development and ripening of *Fragaria* × *ananassa* (cv. Aromas) fruit.

Gene	Days after anthesis (developmental stages)						
	0 (F)	10 (SG)	17 (LG)	20 (W)	21 (T)	23 (50%R)	25 (R)
FaCOI1	0.21	0.08	-0.05	0.00	0.09	-0.04	-0.33
FaMYC2	1.28	0.75	0.29	0.06	0.05	-0.16	-0.54
FaMYC2-like	0.62	0.60	0.14	0.00	-0.30	-0.32	-0.44
FaJAZ1	1.50	0.94	0.59	0.03	-0.25	-1.10	-1.70
FaJAZ4-1/2/3	0.10	0.04	0.00	0.00	-0.03	-0.03	-0.21
FaJAZ5	1.17	0.81	0.43	0.06	-0.17	-0.80	-0.96
FaJAZ7	1.63	0.81	0.62	0.09	-0.40	-1.15	-1.40
FaJAZ8.1	1.47	0.95	1.03	0.07	0.09	-0.77	-0.74
FaJAZ8.2	1.47	0.74	0.84	0.02	-0.05	-0.80	-1.30
FaJAZ9	0.74	0.45	0.40	0.00	0.42	0.17	0.14
FaJAZ10	1.88	1.38	0.69	0.01	-0.23	nd	nd
FaJAZ11	0.66	0.27	0.08	0.01	0.28	0.24	0.11
FaJAZ12	0.76	0.52	0.14	0.00	-0.35	-0.48	-0.59
FaNINJA	0.67	0.50	0.16	0.01	-0.12	-0.20	-0.45
FaTPL1	0.14	-0.04	-0.19	0.01	-0.29	-0.28	-0.68
FaTPL2	0.37	0.06	-0.13	0.00	0.14	0.32	0.22
FaTPL3	0.34	0.10	-0.07	0.01	0.32	0.05	0.24
FaTPL4	0.28	0.16	-0.07	0.00	-0.20	-0.26	-0.53
FaHDA6.1	0.35	0.05	-0.07	0.00	0.13	0.19	0.00
FaHDA6.2	0.26	0.11	0.05	0.00	-0.03	0.03	-0.12
FaHDA19.1	0.33	0.19	0.04	0.01	-0.06	-0.07	-0.19
FaHDA19.2	0.28	0.06	-0.11	0.00	0.06	0.00	-0.10
FaJAM1	1.06	0.62	0.24	0.02	0.03	-0.18	-0.41
FaJAM2	0.12	0.02	-0.16	0.01	-0.14	-0.16	-0.41

Developmental stages correspond to 0 (flowering, F), 10 (small green, SG), 17 (large green, LG), 20 (white, W), 21 (turning, T), 23 (50% red receptacle, 50%R), and 25 (100% red receptacle, R) days after anthesis (DAA). nd = no detection. COI1, coronatine insensitive 1; HDA, histone deacetylases; JAM, jasmonate-associated MYC2-like; JAZ, jasmonate ZIM-domain; NINJA, novel interactor of JAZ; TPL, TOPLESS.
