# Supplementary material for: Jasmonate signalling pathway in strawberry: Genome-wide identification, molecular characterization and expression of JAZs and MYCs during fruit development and ripening
Source: PLoS One. 2018 May 10;13(5):e0197118. doi: 10.1371/journal.pone.0197118 (PMC5944998; doi:10.1371/journal.pone.0197118)
Supplement: S9 Table — Expression levels were measured at 0, 15, 30 min, 1 and 6 h under MeJA treatment. At each treatment and time, three biological replicates were used for the different analysis. Data were analyzed by one-way ANOVA test, and differences among means ± SE (n = 3) were determined using LSD test. Asterisks (*) indicate significant differences between control and MeJA treatment (p ≤ 0.05) for each gene. JAZ, jasmonate ZIM-domain. (PDF) [file pone.0197118.s015.pdf]

S9 Table. Relative expression of *FaMYC2*, *FaJAZ1* and *FaJAZ8.1* in MeJA-treated strawberry (*Fragaria* × *ananassa* cv. Aromas) fruit by RT-qPCR analysis.

Gene	Treatment								
	0 min	15 min		30 min		1 h		6 h	
		Control	MeJA	Control	MeJA	Control	MeJA	Control	MeJA
FaJAZ1	1.01 ± 0.13	1.07 ± 0.24	1.47 ± 0.11	1.72 ± 0.26	3.06 ± 0.82*	1.46 ± 0.28	1.80 ± 0.82	3.65 ± 2.63	2.63 ± 0.89
FaJAZ8.1	1.06 ± 0.39	7.24 ± 1.48	3.25 ± 2.78	12.24 ± 7.95	10.52 ± 0.45	9.74 ± 5.49	7.81 ± 0.06	14.19 ± 2.84	38.50 ± 9.68*
FaMYC2	1.00 ± 0.10	3.48 ± 0.18	6.32 ± 2.07*	2.09 ± 0.47	5.84 ± 1.40*	1.95 ± 0.56	3.26 ± 1.91	1.81 ± 0.17	3.94 ± 0.58*

Expression levels were measured at 0, 15, 30 min, 1 and 6 h under MeJA treatment. At each treatment and time, three biological replicates were used for the different analysis. Data were analyzed by one-way ANOVA test, and differences among means ± SE (n = 3) were determined using LSD test. Asterisks (*) indicate significant differences between control and MeJA treatment ($p \leq 0.05$) for each gene. JAZ, jasmonate ZIM-domain.
