# Supplementary material for: Jasmonate signalling pathway in strawberry: Genome-wide identification, molecular characterization and expression of JAZs and MYCs during fruit development and ripening
Source: PLoS One. 2018 May 10;13(5):e0197118. doi: 10.1371/journal.pone.0197118 (PMC5944998; doi:10.1371/journal.pone.0197118)
Supplement: S10 Table — Expression data were extracted from accession numbers of F. vesca (Sanchez-Sevilla et al. 2017) and we renamed according to the present research gene nomenclature. FaJAZ12 gene was not found in RNAseq experiments from Sanchez-Sevilla et al. (2017). Developmental stages correspond to GA (green achene), GR (green receptacle), RA (ripe achene), RR (ripe receptacle), TA (turning achene), TR (turning receptacle), WA (white achene), WR (white receptacle). COI1, coronatine insensitive 1; HDA, histone deacetylases; JAM, jasmonate-associated MYC2-like; JAZ, jasmonate ZIM-domain; NINJA, novel interactor of JAZ; TPL, TOPLESS. (PDF) [file pone.0197118.s016.pdf]

S10 Table. Original FPKM values of JA signalling-related genes in achene and receptacle during development and ripening of *Fragaria* × *ananassa* (cv. Camarosa) fruit obtained from RNAseq experiments (Sanchez-Sevilla et al. 2017).

Gene	Achene				Receptacle			
	GA_FPKM	WA_FPKM	TA_FPKM	RA_FPKM	GR_FPKM	WR_FPKM	TR_FPKM	RR_FPKM
FaCOI1	31.77	38.22	26.83	24.19	41.87	52.27	64.02	61.81
FaMYC2	34.32	44.86	23.07	7.03	118.89	37.69	28.25	12.37
FaMYC2-like	8.43	0.08	0.04	0.07	14.25	0.06	0.01	0.02
FaJAZ1	114.14	95.92	105.64	69.26	258.17	7.92	2.16	2.76
FaJAZ4-1/2/3	12.64	21.43	21.92	13.00	21.53	25.35	28.32	27.10
FaJAZ5	291.47	143.23	98.53	34.81	702.87	50.17	16.84	36.60
FaJAZ7	9.40	0.57	0.81	0.24	27.54	0.01	0.00	0.06
FaJAZ8.1	102.33	98.17	31.86	35.83	405.02	11.72	8.77	20.05
FaJAZ8.2	7.76	3.20	0.25	0.62	35.87	0.70	0.10	0.25
FaJAZ9	10.89	29.18	47.96	46.87	32.12	13.96	18.56	29.72
FaJAZ10	49.44	7.39	1.54	1.40	107.17	1.18	0.40	2.29
FaJAZ11	2.76	1.24	1.13	1.39	1.44	0.48	0.36	0.84
FaNINJA	30.49	14.60	16.44	13.46	35.17	13.71	14.97	12.52
FaTPL1	24.71	8.09	7.65	11.39	44.06	52.51	33.81	30.18
FaTPL2	27.60	25.06	47.94	67.23	10.68	31.90	50.60	106.98
FaTPL3	30.55	16.34	23.21	40.24	22.50	30.42	34.83	52.66
FaTPL4	33.95	30.60	63.66	38.23	34.22	59.76	311.39	408.60
FaHDA6.1	11.68	26.21	38.29	37.32	8.98	13.73	14.86	18.83
FaHDA6.2	0.00	0.00	0.00	0.00	0.00	0.00	0.00	0.00
FaHDA19.1	62.71	58.51	57.77	67.31	59.02	57.73	58.99	60.09
FaHDA19.2	10.73	6.97	10.45	12.76	9.07	11.82	15.72	14.52
FaJAM1	19.93	6.83	8.20	6.87	17.84	6.29	6.07	4.36
FaJAM2	10.99	5.31	11.76	11.63	11.85	12.50	9.20	6.40

Expression data were extracted from accession numbers of *F. vesca* (Sanchez-Sevilla et al. 2017) and we renamed according to the present research gene nomenclature. *FaJAZ12* gene was not found in RNAseq experiments from Sanchez-Sevilla et al. (2017). Developmental stages correspond to GA (green achene), GR (green receptacle), RA (ripe achene), RR (ripe receptacle), TA (turning achene), TR (turning receptacle), WA (white achene), WR (white receptacle). COI1, coronatine insensitive 1; HDA, histone deacetylases; JAM, jasmonate-associated MYC2-like; JAZ, jasmonate ZIM-domain; NINJA, novel interactor of JAZ; TPL, TOPLESS.
